# Real-world use of ceftolozane/tazobactam: a systematic literature review

**DOI:** 10.1186/s13756-021-00933-8

**Published:** 2021-04-08

**Authors:** Laura Puzniak, Ryan Dillon, Thomas Palmer, Hannah Collings, Ashley Enstone

**Affiliations:** 1 Merck & Co., Inc., 2000 Galloping Hill Road, Kenilworth, NJ 07033 USA; 2 Adelphi Values PROVE, Adelphi Mill, Bollington, Cheshire UK

**Keywords:** Ceftolozane/tazobactam, *Pseudomonas aeruginosa*, Antibacterial resistance, Real-world evidence

## Abstract

**Background:**

Antibacterial-resistant gram-negative infections are a serious risk to global public health. Resistant Enterobacterales and *Pseudomonas aeruginosa* are highly prevalent, particularly in healthcare settings, and there are limited effective treatment options. Patients with infections caused by resistant pathogens have considerably worse outcomes, and incur significantly higher costs, relative to patients with susceptible infections. Ceftolozane/tazobactam (C/T) has established efficacy in clinical trials. This review aimed to collate data on C/T use in clinical practice.

**Methods:**

This systematic literature review searched online biomedical databases for real-world studies of C/T for gram-negative infections up to June 2020. Relevant study, patient, and treatment characteristics, microbiology, and efficacy outcomes were captured.

**Results:**

There were 83 studies comprising 3,701 patients were identified. The most common infections were respiratory infections (52.9% of reported infections), urinary tract infections (UTIs; 14.9%), and intra-abdominal infections (IAIs; 10.1%). Most patients included were seriously ill and had multiple comorbidities. The majority of patients had infections caused by *P.* *aeruginosa* (90.7%), of which 86.0% were antimicrobial-resistant. C/T was used as both a 1.5 g q8h and 3 g q8h dose, for a median duration of 7–56 days (varying between studies). Outcome rates were comparable between studies: clinical success rates ranged from 45.7 to 100.0%, with 27 studies (69%) reporting clinical success rates of > 70%; microbiological success rates ranged from 31 to 100%, with 14 studies (74%) reporting microbiological success rates of > 70%. Mortality rates ranged from 0 to 50%, with 31 studies (69%) reporting mortality rates of ≤ 20%. In comparative studies, C/T was as effective as aminoglycoside- or polymyxin-based regimens, and in some instances, significantly more effective.

**Conclusions:**

The studies identified in this review demonstrate that C/T is effective in clinical practice, despite the diverse group of seriously ill patients, different levels of resistance of the pathogens treated, and varying dosing regimens used. Furthermore, comparative studies suggest that C/T offers a successful alternative to standard of care (SoC).

**Supplementary Information:**

The online version contains supplementary material available at 10.1186/s13756-021-00933-8.

## Background

Antibacterial resistance is a serious risk to global public health. The problem of resistance is especially acute for gram-negative pathogens [1]. Enterobacterales and *Pseudomonas aeruginosa* are the most prevalent gram-negative hospital-acquired infections (HAIs), collectively accounting for 30% of all HAIs in the United States (US) [2]. Patients in intensive care units (ICUs) are particularly vulnerable to gram-negative infections and accounts for 70% of the HAIs acquired in ICUs [2–4].

The burden of infections caused by these pathogens are intensified because of limited effective treatment options. Pathogen susceptibility to many of the available gram-negative antibacterial agents have diminished over time [5]. Patients with infections caused by resistant pathogens have considerably worse outcomes relative to their susceptible counterparts [6, 7]. In a US national database study, patients with multidrug-resistant (MDR) *P. aeruginosa* respiratory infections had higher mortality, an approximately 7-day longer length of stay (LOS), $20,000 excess costs, higher readmission rates, and > $10,000 excess net loss per case for the hospital relative to those with non-MDR *P. aeruginosa* infections [7]. Further, when the infection is caused by resistant pathogens, it increases the likelihood for receipt of initial inappropriate antibacterial therapy, which has been shown to diminish clinical outcomes and increase costs [8, 9].

The challenge of resistance and deleterious impact on outcomes is further compounded by the serious drug-related toxicity associated with some of the current treatment options for resistant gram-negative pathogens. Aminoglycosides (e.g. gentamicin, tobramycin and amikacin) and polymyxins (e.g. colistin) are reported to cause nephrotoxicity and/or ototoxicity [10, 11]. Although these antibacterial agents tend to have higher susceptibility to many gram-negative pathogen, they come at a cost of toxicity.

Due to this imminent threat of drug-resistant Enterobacterales and *P. aeruginosa,* and the limited treatment options and toxic effects of some antibacterial agents, the World Health Organization (WHO) in 2017 designated both Enterobacterales and *P. aeruginosa* as the highest ‘critical’ priority in need of new therapies to counteract this crisis [12].

Ceftolozane/tazobactam (C/T) is a β-lactam/β-lactamase inhibitor antibacterial agent, consisting of a fixed (2:1) combination of an antipseudomonal cephalosporin, ceftolozane, and the well-established β-lactamase inhibitor, tazobactam [13]. C/T is approved in the US and Europe for clinical use in adults with complicated urinary tract infections (cUTIs), including pyelonephritis, complicated intra-abdominal infections (cIAIs), and hospital-acquired bacterial pneumonia/ventilator-associated bacterial pneumonia (HABP/VABP) [13, 14]. The approval of C/T was supported by three multinational, randomized, double-blind, active comparator-controlled trials: ASPECT-cUTI, ASPECT-cIAI and ASEPCT-NP [15–17]. In the ASPECT trials, C/T demonstrated superiority over levofloxacin (ASPECT-cUTI), and noninferiority to meropenem (ASPECT-cIAI and -NP) [15–17]. Since launch in 2014, real-world evidence (RWE) for the use of C/T in clinical practice has been accumulating. The purpose of this systematic literature review (SLR) was to identify and collate published RWE to better understand the characteristics of patients treated with C/T and clinical outcomes.

## Methodology

### Literature search

A search of the literature for C/T RWE, published between 1st January 2009 and 3rd June 2020, was conducted in the following biomedical and economic databases via the OVID platform: Embase, MEDLINE, PsycInfo, Econlit, and EBM Reviews (ACP Journal Club, Cochrane Database of Systematic Reviews, Cochrane Methodology Register, Database of Abstracts of Reviews of Effects, Health Technology Assessment, NHS Economic Evaluation Database, Cochrane Clinical Answers). The search was conducted in January 2019 with a 10-year time horizon, then re-ran to capture literature published between January–November 2019, and November 2019–June 2020. The time horizon was chosen to minimize erroneous data identification given that C/T was approved for use in 2014—using a longer horizon would capture any publications reporting on expanded access or compassionate use. The search was limited to English Language publications only.

Due to the heterogeneity of reporting of RWE, the search was designed to be broad to ensure relevant studies which may not be appropriately indexed were retrieved. Table 1 details the search strategy.

**Table 1:** OVID search strategy

#	Search terms
1	Ceftolozane/ OR Ceftolozane plus tazobactam/
2	((Ceftolozane adj1 tazobactam) OR ZERBAXA OR MK-7625A).ti,ab
3	1 OR 2
4	(exp animals/ OR nonhuman/) NOT exp human/
5	exp controlled clinical trial/
6	4 OR 5
7	3 NOT 6
TOTAL (deduplicated and limits* applied)	

^*^English and 2009–current

A further search of internet-based sources relating to C/T RWE was also conducted (limited to English language only). This gray literature review involved searching conference proceedings of two conferences—European Congress of Clinical Microbiology and Infectious Diseases [ECCMID] and Infectious Disease Week (IDWeek)—two of the largest infectious disease conferences in Europe and the US. Conference proceedings, when published as part of an abstract book, were also identified during the OVID search.

### Study selection

All screening (by title and abstract, and by full-text) was performed by two reviewers and any uncertainties were resolved by a third reviewer. Predetermined inclusion and exclusion criteria were used to assess the eligibility of identified abstracts and full-texts for inclusion. PICOS eligibility criteria for study inclusion included observational and non-controlled studies reporting on the use of C/T to treat adult patients (≥ 18 years of age) with gram-negative infections in real-world clinical practice. Only studies in English were included. Studies were excluded if they did not meet the PICOS criteria, such as randomized controlled trials (RCTs) or other randomized or controlled experimental studies. A complete description of the PICOS criteria is provided in Additional file 1: Table S1.

### Data extraction and analysis

Relevant study, patient, and treatment characteristics, microbiology, and efficacy outcomes were extracted into a data extraction form by one reviewer and checked by a senior reviewer. Efficacy outcomes included clinical cure (typically defined as the resolution of signs or symptoms of infection following therapy and survival), microbiological cure (typically defined as large reduction or eradication in the number of pathogens following therapy), and mortality.

## Results

### SLR results

**Fig. 1:**
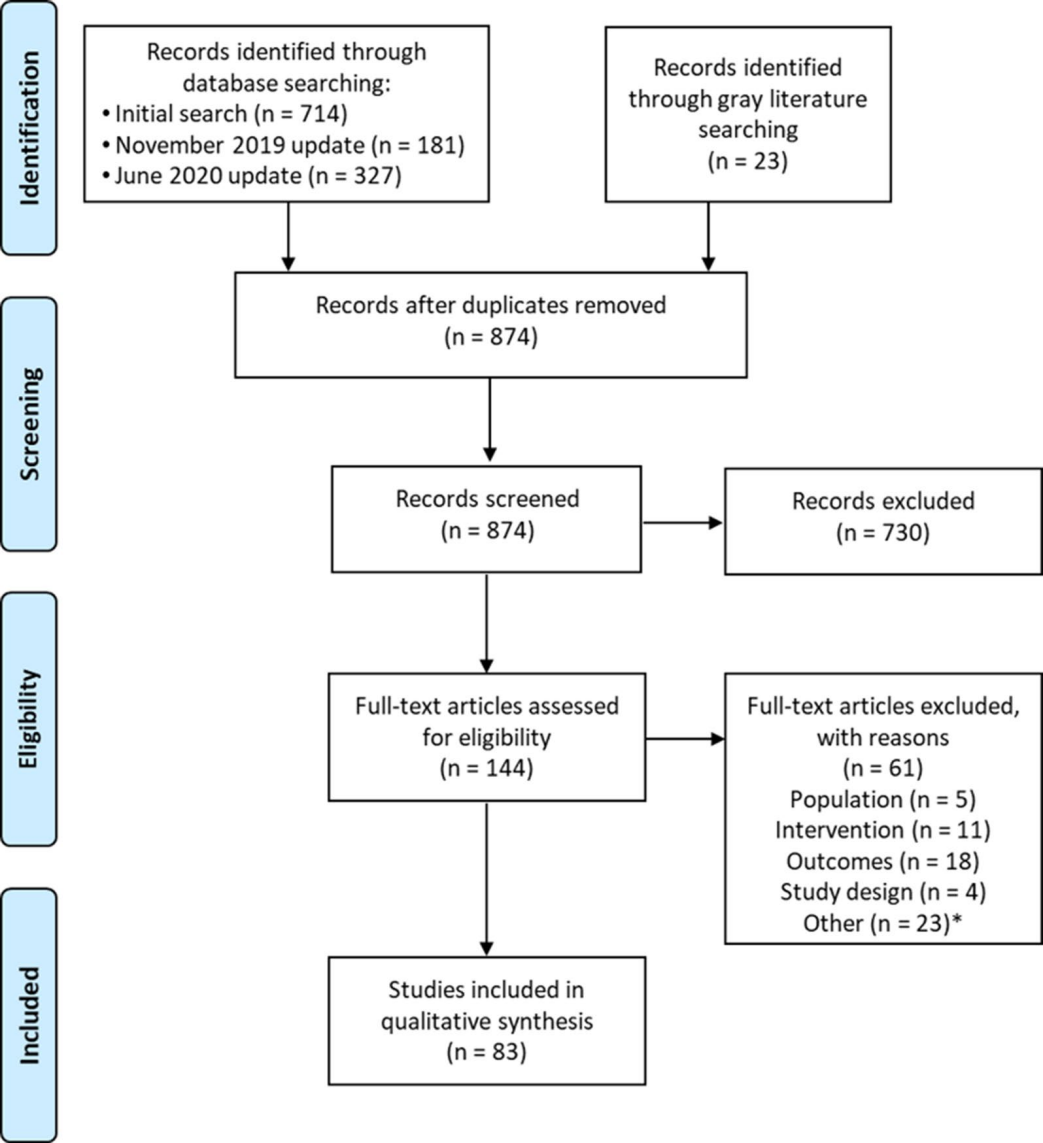
PRISMA flow diagram for study selection. * ‘Other’ includes the exclusion of duplicate records

A total of 1,222 records were identified from the database searches, and 23 records were identified from the gray literature search. This resulted in 874 non-duplicate records that were subject to title and abstract screening. A total of 730 records were excluded according to the PICOS criteria and 144 were included for full-text review. Of these, 83 studies were determined to be eligible for data extraction and qualitative synthesis. The results of the SLR and study selection processes are presented in Fig. 1.

### Study characteristics

Of the 83 studies included in the SLR, 61 were published as peer-reviewed publications [18–78], and 22 were conference proceedings (availability as abstracts or posters) [79–100]. Including studies that recruited patients from multiple countries, the most common study locations were the US (N = 50), [21, 22, 24, 27–29, 33, 34, 39–41, 43–45, 50, 51, 54, 56, 57, 59, 61, 62, 68–71, 73–77, 79–85, 87–92, 94, 95, 97–100] Spain (N = 15) [26, 28, 30–32, 35–37, 42, 47, 49, 58, 66, 79, 96], and Italy (N = 13) [18, 20, 23, 25, 48, 52, 53, 55, 64, 67, 72, 79, 86]. A variety of study designs were captured: 27 were non-comparative retrospective studies [18, 19, 22, 24, 25, 28, 32, 33, 40, 41, 79, 81, 84–92, 94–99], 14 were case series [20, 21, 29, 31, 34–39, 42, 43, 82, 100], five were comparative (including two cohort studies [80, 83], and three case–control studies [23, 26, 27]) and one was a non-comparative prospective study [30]. There were thirty-six single-patient case reports identified [44–78, 93]. Case reports were included to capture uses of C/T in special clinical situations. Additional file 1: Table S2 in the supplementary material summarizes the single-patient case reports identified by the SLR. There were 47 studies (24 multicenter) reporting on more than one patient, as summarized in Table 2 [18, 22, 23, 25, 27, 28, 33, 37, 38, 40, 79–81, 83–85, 87–91, 94, 97, 99].

**Table 2:** Summary of included studies

Citation, study design, location	N C/T	Patient/infection description	Disease severity	C/T treatment	Outcome, % (n/N)		
					Clinical	Micro	Mortality
*2020 Studies*							
Peer-reviewed literature							
Bassetti et al. 2020 [18] Retrospective, multicenter Italy	153	ESBL-producing Enterobacterales infections, including NP (30.0%), cUTI (22.2%), and cIAI (16.3%)	ICU N = 74 CCI mean = 4.9	Dose C/T: 1.5 g q8h (75.0; of which 6 patients received creatinine clearance adjusted dose) or 3 g q8h (24.8%) Empiric C/T: 30.0% Confirmed C/T: 70.0% Duration: med. (range): 14 (8–25) days	83.7 (128/153)	–	9.8 (15/153)
Bosaeed et al. 2020 [19] Retrospective, single center Saudi Arabia	19	MDR PsA infections, including NP (32%), CLABSI (21%), and ABSSSI (16%), and cIAI (16%)	ICU N = 12	Dose C/T: 1.5 g q8h (42.1%) or 3 g q8h (10.5%) or creatinine clearance adjusted (47.4%) Duration: med. (range): 14 (7–35) days	95 (18/19)	74 (14/19)	21 (4/19)
Buonomo et al. 2020 [20] Retrospective, single center case series Italy	4	PsA (50% MDR; 50% XDR) cSSTI in patients with chronic kidney disease	-	Dose C/T: creatine clearance adjusted (100.0%)—0.75 g q8h (75%), 0.375 g q8h (25%) Empiric C/T: 0.0% Confirmed C/T: 100.0% Duration: med. (range): 14 (14) days	100.0 (4/4)	-	0
Jones et al. 2020 [21] Retrospective single center, case series US	7	PsA (57.1% non-MDR; 42.9% MDR) infections (one patient also had an *E. coli* infection), including pneumonia (42.9%), cUTI (28.6%), and bacteremia (14.3%)	–	Dose C/T: 4.5 g qd (CI; 85.7%), 9 g qd (CI; 14.3%) Duration: med. (range): 14 (6–42) days	85.7 (6/7)	100.0 (3/3)	0^a^
Jorgensen et al. 2020 [22] Retrospective, multicenter US	259	MDR gram-negative infections (91.1% PsA; 23.2% Enterobacterales) including, RTIs (62.9%), SSTIs (10.8%), and UTIs (10.0%). Patients with MDR PsA infections (N = 226) were used as the primary analysis set	ICU N = 131 IMC N = 23 APACHE II med. = 21 CCI med. = 3 SOFA med. = 5	Dose C/T: 1.5 g q8h (36.3%) or 3 g q8h (63.7%), creatinine clearance adjusted (30.5%) Duration: med. (IQR): 10 (6–15) days	MDR PsA (N = 226) *Clinical failure:* 37.6 (85/226)	-	MDR PsA (N = 226) 17.3 (39/226)
Vena et al. 2020 [23] Retrospective, multicenter, case–control Italy	16	Drug-resistant PsA (62.5% MDR; 37.5% XDR) pneumonia and bacteremia	ICU N = 2	Duration: mean (SD): 12.1 (5.8) days	81.3 (13/16)	-	18.8 (3/16)
Conference proceedings							
Caffrey et al. 2020 [80] Retrospective, multicenter, cohort US	57	MDR PsA infections, including RTIs (36.8%), UTIs (22.8%), and SSTIs (17.5%)	ICU N = 36 APACHE II med. = 40 CCI med. = 4	Duration med. (IQR): 12 (5–18) days	-	31.0 (13/42)	17.5 (10/57)
Gudiol et al. 2020 [79] Retrospective, multicenter International	31	PsA (90.3% MDR; 41.9% XDR) bloodstream infections in neutropenic cancer patients	ICU N = 7 IMC N = 31	Empiric C/T: 25.8% Confirmed C/T: 96.8%	-	-	16.1 (5/31)
*2019 studies*							
Peer-reviewed literature							
Bassetti et al*.* 2019 [25] Retrospective, multicenter Italy	101	PsA (70% drug resistant) infections, including NP (31.7%), ABSSSI (20.8%), and cUTI (13.9%)	ICU N = 24 CCI mean = 4.4	Dose C/T: 1.5 g q8h (69.3%) or 3 g q8h (30.7%) Duration: med. (range): 14 (9–23) days	83.2 (84/101)	-	5.0 (5/101)
Fernández-Cruz et al. 2019 [26] Retrospective, single center, case–control Spain	19	PsA (52.6% MDR; 47.4% XDR) infections, including pneumonia (26.3%), catheter-related BSI (21.1%), and primary BSI (21.1%) in patients with hematological malignancy	ICU N = 5 IMC N = 19 CCI mean = 3.0 SOFA mean = 5.4	Empiric C/T: 15.8% Confirmed C/T: 84.2% Duration: med. (range): 14 (7–18) days	89.5 (17/19)	-	5.3 (1/19)
Gerlach et al*.* 2019 [24] Retrospective, single center US	18	MDR PsA osteomyelitis	ICU N = 11 APACHE II med. = 13.5 CCI med. = 5.5	Dose C/T: 1.5 g q8h (27.7%) or 3 g q8h (55.6%), or creatinine clearance adjusted (16.7%) Empiric C/T: 0.0% Confirmed C/T: 100.0% Duration: med. (range): 39 (3–98) days	50.0 (9/18)	75.0 (3/4)	22.2 (4/18)
Pogue et al. 2019 [27] Retrospective, multicenter, case–control US	100	MDR or XDR PsA infections, including NP (VABP [52.0%], HABP [12.0%]), cUTIs (16.0%), and wound (13.0%)	ICU N = 70 IMC N = 14 CCI mean = 3 SOFA = 8	Dose C/T: 3 g q8h (63%), 1.5 g q8h (38%) Duration: med. (IQR): 9.5 (7–14) days	81.0 (81/100)	-	20.0 (20/100)
Rodriguez-Nunez et al. 2019 [28] Retrospective, multicenter International	90	Drug-resistant PsA RTIs (76.7% XDR; 23.3% MDR)	CCI med. = 5	Dose C/T: standard (1.5 g q8h or creatinine clearance adjusted; 40%), high (3 g q8h or double creatinine clearance 60%) Duration: med. (IQR): 14 (10–16) days	56.7 (51/90)	-	27.8 (25/90)
Tan et al. 2019 [29] Retrospective, single center, case series US	5	MDR gram-negative (60% PsA; 40% *A. baumannii*) osteomyelitis	-	Dose C/T: 1.5 g q8h (20%), 3 g q8h (80%) Empiric C/T: 0% Confirmed C/T: 100% Duration mean: 37.8 days	60.0 (3/5)	-	20.0 (1/5)
Conference proceedings							
Cabrera et al*.* 2019 [85] Retrospective, multicenter US	45	Gram-negative (84.4% PsA; 71.1% MDR PsA) infections, including pneumonia (38%), UTI (20%), wound (9%), and bone (9%)	ICU N = 19 IMC N = 6	Empiric C/T: 21.7% Confirmed C/T: 78.3% Duration med. (IQR): 8 (4–12) days	68.9 (31/45)	-	0
Hart et al*.* 2019 [84] Retrospective, multicenter US	70	MDR PsA infections, including pneumonia (56%), wound (11%), IAI (10%) in immunocompromised patients	ICU N = 33 IMC N = 70 APACHE II med. = 18 CCI med. = 5	Duration mean (SD): 13 (10.8) days	69 (48/70)	-	19 (13/70)
Mills et al*.* 2019 [83] Retrospective, multicenter cohort US	62	MDR PsA pneumonia	ICU N = 49 IMC N = 13	Duration mean: 16.1 days	72.6 (45/62)	-	29 (18/62)
Sheffield et al. 2019 [82] Retrospective, case series US	4	PsA or ESBL-producing *E. coli* infections, including LVAD infection (50.0%), RTI (25.0%), and IAI (25.0%)	-	Dose C/T med.: 6 g CI qd Duration range: 6–91 days	-	-	0
Trisler et al*.* 2019 [81] Retrospective, multicenter US	35	PsA infections, including RTI (71.4%), IAI (14.3%), and osteomyelitis (5.7%) in patients with and without CF	-	Empiric C/T: 0.0% Confirmed C/T: 100.0% Duration med. (IQR): CF = 18.5 (14–37.5) days, non-CF = 15.0 (10–25) days	*Clinical failure:* 54.3 (19/35)	-	-
*2018 studies*							
Peer-reviewed literature							
Diaz-Cañestro et al*.* 2018 [30] Prospective, single center Spain	58	PsA (86.2% XDR) infections, including RTIs (60.3%), UTIs (17.2%), and IAIs (6.9%)	ICU N = 16 IMC N = 7 CCI med. = 4 SOFA med. = 3	Dose C/T: 1.5 g q8h (46.6%), 3 g q8h (41.4%), 0.75 g q8h (12.1%) Empiric C/T: 1.7% Confirmed C/T: 91.4% Duration mean (SD): 11.4 (6.2) days	63.8 (37/58)	-	27.6 (16/58)
Dietl et al*.* 2018 [31] Retrospective, single center, case series Spain	7	XDR PsA SSTIs (43%) and osteomyelitis (57%)	CCI med. = 6	Dose C/T: 1.5 g q8h (43%), 0.75 g q8h (29%), 0.375 g q8h (29%) Empiric C/T: 0% Confirmed C/T: 71% Duration med. (range): SSTI 13 (4–27)/ osteo. 48 (21–66) days	86 (6/7)	100 (4/4)	0
Escolà-Vergé et al. 2018 [32] Retrospective, single center Spain	38	XDR PsA infections, including RTIs (36.8%), SSTIs (15.8%), and UTIs (15.8%)	ICU N = 12 CCI med. = 3.5	Dose C/T: 3 g q8h (60.5%), 1.5 g q8h (39.5%) Duration med. (range): 15.5 (3–62) days	68.4 (26/38)	*Micro. recur.:* 31.6 (12/38)	13.2 (5/38)
Gallagher et al. 2018 [33] Retrospective, multicenter US	205	MDR PsA infections, including 59% pneumonia, UTI (13.7%), and wound (12.7%)	ICU N = 105 APACHE II med. = 19 CCI med. = 4	Dose C/T: 3 g q8h (47.3%), 1.5 g q8h (52.7%) Duration med. (IQR): 10 (7–14) days	73.7 (151/205)	70.7 (145/205)	19.0 (39/205)
Hakki et al. 2018 [34] Retrospective, single center, case series US	6	7 episodes of MDR PsA infections, including bacteremia (42.9%), pneumonia (42.9%), and soft tissue (14.3%) in patients with hematological malignancy or hematopoietic stem cell transplant	IMC N = 6	Dose C/T: 3 g q8h (100%) Empiric C/T: 33.3% Confirmed C/T: 66.7% Duration med. (range): 29 (14–103) days	71.4 (5/7)^b^	-	0
Xipell et al. 2018 [35] Retrospective, single center, case series Spain	23	24 episodes of MDR PsA infections, including RTI (33.3%), UTI (29.2%). and SSTI (25.0%)	ICU N = 4	Dose C/T: 3 g q8h or 1.25 g q8h or 0.75 g q8h (% = NR) Empiric C/T: 13% Confirmed C/T: 87% Duration mean (SD): 14.3 (9.4) days	88 (21/24)	75 (12/16)	22 (5/23)
Conference proceedings							
Elabor et al. 2018 [97] Retrospective, multicenter US	65	MDR PsA infections, including pneumonia, wound/bone/joint infections, UTIs, and IAIs (% NR) in immunocompromised patients	ICU N = 37 IMC N = 65 APACHE II med. = 20 CCI med. = 6	Dose C/T: 3 g q8h (35.4%), 1.5 g q8h (35.4%), < 1.5 g q8h (29.2%)	78.4 (51/65)	75.3 (NR)	13.9 (9/65)
Gioia et al*.* 2018 [96] Retrospective, single center Spain	15	MDR PsA infections, including RTI (53%), IAI (27%), and wound (13%)	ICU N = 8 IMC N = 9 CCI med. = 4	Dose C/T: 1.5 g q8h (67%), < 1.5 g q8h (13%), 3 g q8h (20%) Duration med. (range): 23 (2–102) days	60 (9/15)	60 (9/15)	27 (4/15)
Henry et al. 2018 [95] Retrospective, single center US	29	42 treatment courses for gram-negative infections (86% PsA; 7% *Klebsiella* spp.; 7% *E. coli*), including pneumonia (26%), IAIs (21%), and UTI (21%)	ICU N = 15	Dose C/T: med. (range) = 1.5 g (0.15–3 g) q8h Empiric C/T: 36% Confirmed C/T: 64% Duration med. (range): 10 (2–85) days	76 (32/42)	-	38 (11/29)
Hirsch et al. 2018 [94] Retrospective, multicenter US	35	Gram-negative infections (79% PsA: 60.7% MDR; 21.4% XDR), including RTIs (33%), BSIs (21%), and bone/joint infections (18%)	ICU N = 26	Dose C/T: 3 g q8h (42.9%), 1.5 g q8h (31.4%), 0.75 g q8h (17.1%), 0.375 g q8h (2.9%), Other (5.7%) Empiric C/T: 20% Confirmed C/T: 80%	77.4 (24/31)	74.2 (23/31)	14.3 (5/35)
Jayakumar et al*.* 2018 [92] Retrospective, single center US	22	PsA (95%; 90% MDR) sepsis and/or bacteremia infections	-	Dose C/T: 3 g q8h (55%), Other (45%) Empiric C/T: 18% Confirmed C/T: 82% Duration med.: 10 days	77 (17/22)	-	23 (5/22)
Jorgensen et al*.* 2018 [90] Retrospective, multicenter US	116	MDR PsA infections, including RTI (65%), UTI (10.3%), and SSTI (9.4%)	ICU N = 72 IMC N = 22 APACHE II med. = 21 CCI med. = 3.5	-	*Clinical failure:* 38.8 (45/116)	-	17.2 (20/116)
Jorgensen et al*.* 2018 [91] Retrospective, multicenter US	137	MDR PsA infections	ICU N = 87 IMC N = 11	-	-	-	18.2 (25/137)
Pogue et al*.* 2018 [89] Retrospective, multicenter US	113	PsA cUTI (64%) and cIAI (36%)	-	Empiric C/T: 31% Confirmed C/T: early definite 28% and late definite 41%	-	-	12.4 (14/113)
Puzniak et al. 2018 [87] Retrospective, multicenter US	1,490	Gram-negative infections (78% PsA [202/259 patients with microbiological results])	ICU N = 824 CCI mean = 3	-	-	-	9.1 (NR)
Puzniak et al. 2018 [88]^c^ Retrospective, multicenter US	199	PsA infections, including RTIs (57%) and UTIs (17%)	ICU N = 107 CCI mean = 2.9	Empiric C/T: 34% Confirmed C/T: early direct 50% and late direct 16% Duration med. (IQR): 8 (4–13) days	-	-	14 (28/199)
Tordato et al*.* 2018 [86] Retrospective, single center Italy	11	PsA infections (73% XDR), including RTIs (54%), BSIs (27%), and IAIs (18%)	ICU N = 6 IMC N = 3 CCI med. = 4	Duration med. (range): 16 (6–27) days	100.0 (11/11)	-	36.4^d^ (4/11)
*2017 studies*							
Peer-reviewed literature							
Álvarez Lerma et al. 2017 [36] Retrospective, single center, case series Spain	2	PDR PsA ventilation-associated respiratory infections	ICU N = 2 APACHE II mean = 25.5	Dose C/T: 1.5 g q8h then 0.75 g q8h (50%), 0.75 g q8h (50%) Empiric C/T: 0% Confirmed C/T: 100% Duration: mean = 15.5 days	100 (2/2)	100 (2/2)	50 (1/2)
Castón et al. 2017 [37] Retrospective, multicenter, case series Spain	12	MDR PsA infections, including RTIs (50%) and IAIs (25.0%). 83% of patients had septic shock	IMC N = 4	Dose C/T: 1.5 g q8h (67%), 3 g q8h (33%) Empiric C/T: 0% Confirmed C/T: 100% Duration med. (range): 12 (9–18) days	75.0 (9/12)	63.6 (7/11)	25.0 (3/12)
Dinh et al. 2017 [38] Retrospective, multicenter, case series France	15	XDR PsA infections, including RTIs (46.7%), UTIs (20.0%), and IAIs (13.3%)	ICU N = 8 IMC N = 10 SOFA mean = 7.6	Dose C/T: med. (range) = 6 g (3–7.5 g) Duration med. (range): 15 (4–63) days	67 (10/15)	75 (6/8)	27 (4/15)
Haidar et al*.* 2017 [39] Retrospective, single center, case series US	21	MDR PsA infections, including 86% RTIs, 5% cUTIs, 5% cIAIs, and 5% bacteremia	IMC N = 9 CCI med. = 5 SOFA med. = 6	Dose C/T: 1.5 g q8h (48%), 0.75 g q8h (24%), 0.375 g q8h (5%), Other (23%) Duration med. (range): 14 (3–52) days	*Clinical failure:* 29 (6/21)	-	10 (2/21)
Munita et al*.* 2017 [40] Retrospective, multicenter US	35	CR PsA infections, including pneumonia (51.0%) and secondary BSI (17.1%)	CCI med. = 4	Dose C/T: 3 g q8h (26%), 0.375–1.25 g q8h (% = NR) Duration med. (range): 16 (5–27) days	74 (26/35)	100 (25/25)	22.8 (8/35)
Sacha et al. 2017 [41] Retrospective, single center US	49	60 courses of therapy for gram-negative infections (86.7% PsA: 34.6% non-MDR; 40.4% MDR; 25.0% XDR), including NP (56.7%), IAI (18.3%), and bacteremia (6.7%)	ICU N = 37 IMC N = 25	Dose C/T: 3 g q8h (1.7%), 1.5 g q8h (51.7%), 0.75 g q8h (26.7%), 0.375 g q8h (8.3%), 0.15 g q8h (11.7%) Empiric C/T: 36.7% Confirmed C/T: 63.3% Duration med.: 1–8 days^e^	64.1^f^ (25/39)	38.5 (5/13)	16.7 (10/60)
Xipell et al. 2017 [42] Retrospective, single center, case series Spain	3	MDR or XDR PsA infections, including mediastinitis, liver abscess, and septic shock	-	Dose C/T: 1.5 g q8h (100%) Empiric C/T: 0% Confirmed C/T: 100% Duration mean (range): 30.3 (21–42) days	100 (3/3)	-	0
Conference proceedings							
Leuthner et al. 2017 [98] Retrospective, single center US	30	Gram-negative infections (93% PsA; 3% *E. coli*; 3% *P. stuartii*), including RTIs (67%), cUTIs (27%), and BSIs (20%)	ICU N = 8 IMC N = 4	Dose C/T: 3 g q8h (57%), Other (43%) Empiric C/T: 23% Confirmed C/T: 77% Duration med.: 10 days	80 (24/30)	92 (11/12)	20 (6/30)
*2016 studies*							
Conference proceedings							
Iovleva et al*.* 2016 [100] Retrospective, single center, case series US	2	Imipenem-resistant PsA HCAP	APACHE II mean = 13 CCI mean = 2	-	100 (2/2)	100 (2/2)	0
Nathan et al*.* 2016 [99] Retrospective, multicenter US	28	Gram-negative infections (68% resistant pathogens, including 36.4% MDR PsA and 15.2% ESBL-producing *E. coli*), including RTI (28.6%), cIAI (25%), and cUTI (25%)	ICU N = 0	Duration: med. = 12 days for RTI, 12 days for cIAI and 15 days for cUTI	89 (24/27)	-	-
*2015 studies*							
Peer-reviewed literature							
Gelfand et al*.* 2015 [43] Retrospective, single center, case series US	3	MDR PsA pneumonia	IMC = 2	Dose C/T: 3 g q8h (100%) Duration mean (range): 12.7 (10–14) days	100 (3/3)	100 (3/3)	0

^a^2 patients died—both completed therapy and were in the clinical cure group, but later succumbed to comorbid conditions

^b^2 of the 7 courses were considered clinical failures. One patient with clinical failure then had a successful C/T course

^c^This study contains a subset of patients identified in Puzniak et al. 2018 [87]

^d^Although all patients had a favorable clinical outcome, 4 patients were reported to have died from other causes

^e^Median duration of therapy in patients who received pathogen-directed therapy was 8 days; empiric-turned-pathogen-directed therapy, 8 days; empiric-remained-empiric therapy, 7.5 days; and empiric therapy that was subsequently changed or discontinued, 1 day

^f^Only assessed in patients with C/T-susceptible infections

ABSSSI: Acute bacterial skin and skin structure infection; APACHE: Acute Physiology and Chronic Health Evaluation; BSI: Bloodstream infection; CCI: Charlson Comorbidity index; CI: Continuous infusion; cIAI: Complicated intra-abdominal infection; CF: Cystic fibrosis; CLABSI: Central-line-associated bloodstream infection; CR: Carbapenem-resistant; cSSTI: Complicated skin and soft tissue infection; C/T: Ceftolozane/tazobactam; cUTI: Complicated urinary tract infection; ESBL: Extended-spectrum β-lactamase; HABP: Hospital-acquired bacterial pneumonia; HCAP: Healthcare-associated pneumonia; IAI: Intra-abdominal infection; ICU: Intensive care unit; IMC: Immunocompromised; IQR: Interquartile range; LVAD: Left-ventricular assist device; MDR: Multidrug-resistant; NP: Nosocomial pneumonia; NR: Not reported; PDR: Pandrug-resistant; PsA: *Pseudomonas aeruginosa*; RTI: Respiratory tract infection; SD: Standard deviation; SOFA: Sequential Organ Failure Assessment; SSTI: Skin and soft tissue infection; US: United States; UTI: Urinary tract infection; VABP: Ventilator-associated bacterial pneumonia; XDR: Extensively-drug-resistant

### Patient characteristics

**Fig. 2:**
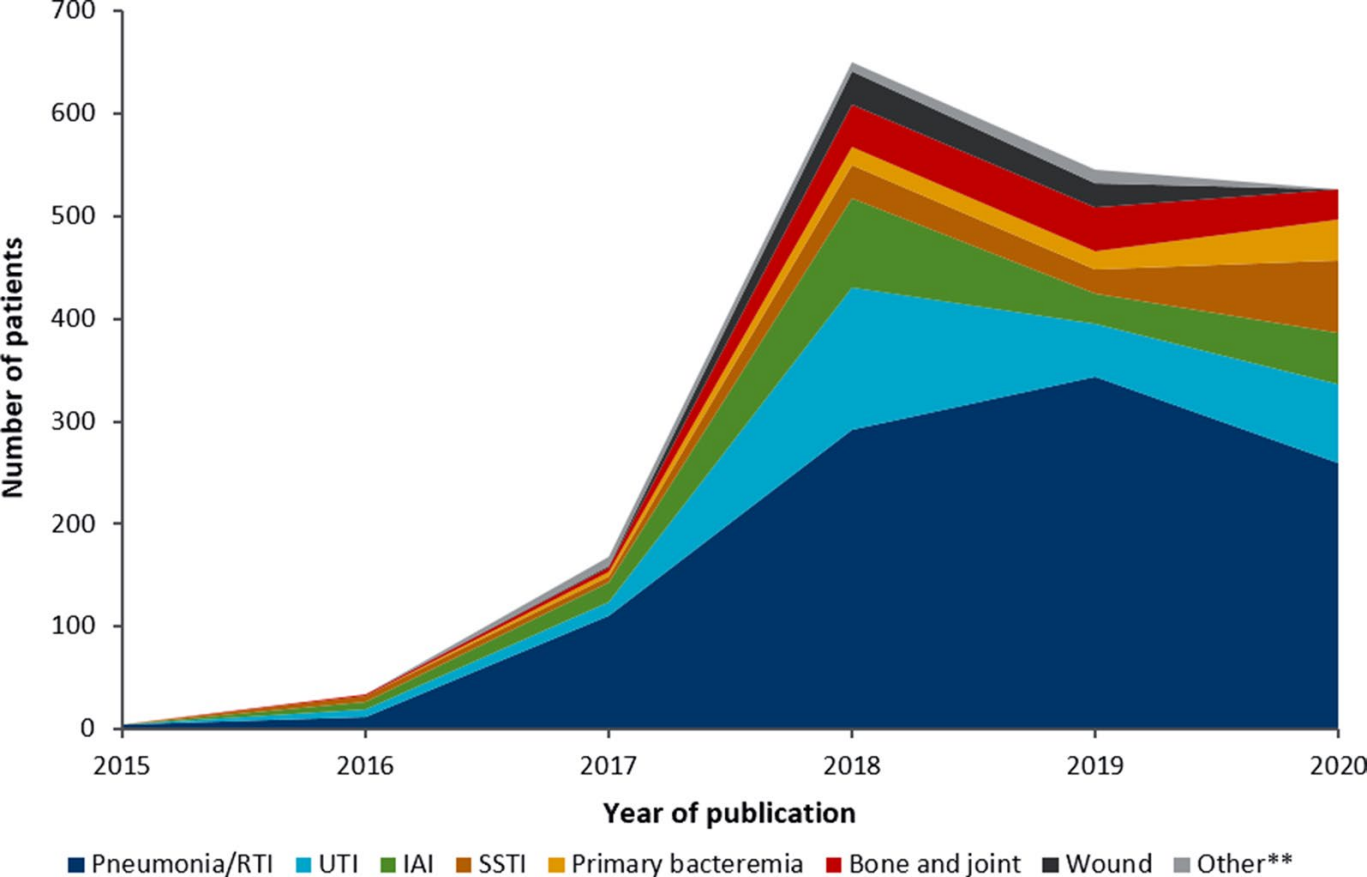
Infections of patients treated with C/T by publication year*. *Excluding patients for which infection was not specified. **Other includes genital infection, CNS infection, liver abscess, mediastinitis, device-related infections, vascular infection, and otitis and mastoiditis. CNS: Central nervous system; C/T: Ceftolozane/tazobactam; IAI: Intra-abdominal infection; RTI: Respiratory tract infection; SSTI: Skin and soft tissue infection; UTI: Urinary tract infection

Identified studies included a total 3701 distinct patients treated with C/T. Excluding the single-patient case reports, the median number of patients included was 30 (range: 2(100)–1490(87)). Patient populations were heterogeneous, with a number of different sources of infections and pathogens reported. There were 3,735 total infections. Of these, there were 1807 infections where the source of infection was not reported (48.4%); excluding those publications, the most common source of infection(s) were pneumonia/respiratory tract infections (RTIs; 52.9% of reported infections), UTIs (14.9%), and IAIs (10.1%). There was also report of C/T use in SSTIs (7.1%), bone and joint infections (6.1%), and primary bacteremia (4.2%). Over time, the number of patients treated with C/T has grown, but the proportion of each infection type has remained relatively consistent (Fig. 2). The number of patients treated for RTIs was consistently high over the time period studied (Fig. 2)—100.0% of identified patients treated with C/T in 2015, 35.3% in 2016, 65.5% in 2017, 44.9% in 2018, 62.9% in 2019, and 49.1% in 2020 had RTIs, and the number of patients treated with C/T for these infections has grown year-on-year.

The patient population included in these RWE publications were often classified as seriously ill with multiple comorbidities. In total, 1,751 patients (47.3% of 3,701 patients reported) were admitted to the ICU. The literature review recorded three commonly used measures of patient illness severity—Acute Physiology and Chronic Health Evaluation (APACHE) II, Sequential Organ Failure Assessment (SOFA), and Charlson Comorbidity (CC) index. APACHE and SOFA are systems for predicting ICU mortality. Nine publications, comprising 794 patients treated with C/T, reported APACHE scores ranging from 13 to 40, with larger studies (> 50 patients) ranging from 18 to 40 [22, 24, 33, 36, 80, 84, 90, 97, 100]. Six publications, comprising 472 patients treated with C/T, reported SOFA scores ranging from 3 to 8 [22, 26, 27, 30, 38, 39]. The CC index quantifies the comorbidity burden of included patients by predicting the mortality of patients with multiple comorbidities. Twenty-one publications, comprising 2930 patients, reported CC index scores ranging from 2 to 6 [18, 22, 24–28, 30–33, 39, 40, 80, 84, 86, 87, 90, 96, 97, 100]. These measures show the high severity of illness of patients included in the RWE of C/T treatment.

Furthermore, this review identified 30 publications reporting a total of 364 immunocompromised patients [22, 26, 27, 30, 34, 37–39, 41, 43, 48, 49, 51, 53, 59–61, 63, 68, 73, 79, 83–86, 90, 91, 96–98]. Immunocompromised patients include those with a history of organ transplant, disease suppressing immunity (e.g. human immunodeficiency virus [HIV]/acquired immunodeficiency syndrome [AIDS], lymphoma, leukemia), receipt of chemotherapy, or immunosuppressive treatment (e.g. corticosteroids). Of these studies, 5 reported only immunocompromised patients [26, 34, 43, 79, 84].

**Fig. 3:**
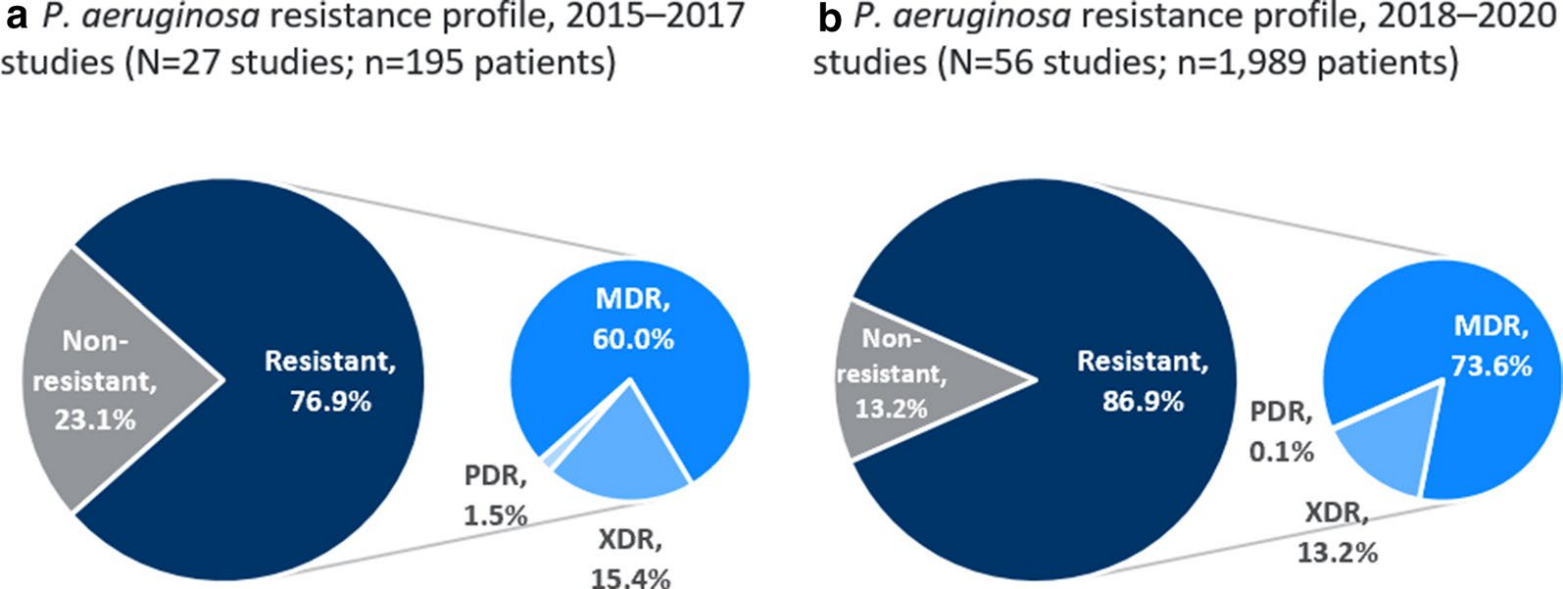
*P. aeruginosa* resistance profile in studies identified in 2015–2017 and 2018–2020. MDR: Multidrug-resistant; PDR: Pandrug-resistant; XDR: Extensively-drug-resistant

A total of 1,294 (35.0%) patients did not have a causative pathogen specified (note that the majority of these came from a single publication [87]. For publications that reported a causative pathogen, the majority of patients (90.7%; N = 2,184) had infections that were caused by *P. aeruginosa*, of which 14.0% were caused by non-drug resistant *P. aeruginosa*, or the level of resistance was not specified, 72.3% by MDR *P. aeruginosa*, 13.4% extensively-drug-resistant (XDR), and 0.2% pan-drug-resistant (PDR). Note that the level of resistance specified (MDR/XDR/PDR) was recorded as described in the publication. Resistant infections comprised the majority of infections treated in studies published in the first three-year period captured (2015–2017) vs. the second three-year period (Fig. 3).

### Treatment characteristics

C/T is indicated for use at two doses: either 1.5 g q8h (for cIAI and cUTI) or 3 g q8h (for patients with HABP/VABP). For patients with renal insufficiency, doses are reduced according to level of creatinine clearance. In studies that reported dosing information (N = 1,418 patients), C/T was used as a 1.5 g q8h dose in 619 (43.7%) patients, as a 3 g q8h dose in 621 (43.8%) patients, and as a creatinine clearance adjusted dose in 178 (12.6%) patients. Note, however, that reporting of dosing was inconsistent between studies and the specific dose by type of infection (i.e., 3 g q8h for respiratory) was not always delineated. Of studies that reported the timing of C/T treatment (N = 893 patients), C/T was administered empirically (i.e. prior to susceptibility results) in 222 (24.9%) patients and administered confirmed (i.e. following susceptibility results) in 671 (75.1%). There was little year-on-year change in the proportion of patients treated empirically or confirmed, or treated with a 1.5 g q8h or 3 g q8h regimen—despite the approval of the 3 g q8h dose in 2019.

There was large variation in the duration of C/T therapy reported, often different to the label dose of 4–14 (cIAI), 7 (cUTI), or 8–14 (HABP/VABP) days. In all studies, the median duration of C/T therapy ranged from 7 to 56 days, irrespective of dose. Median duration in larger studies (> 50 patients) ranged from 8 to 16.1 days, consistent with the indicated duration. Excluding single-patient case reports, 12 studies (231 patients) reported an average duration of C/T exceeding the label maximum dose of 14 days; with three studies (26 patients) reporting an average duration of > 28 days. Of these three studies, two included patients with osteomyelitis and MDR *P. aeruginosa* infection [24, 29], and one included patients with severe infections caused by MDR or XDR *P. aeruginosa* [42]. Furthermore, 15 single-patient case reports reported C/T durations exceeding the maximum label dose, with 12 reporting a duration of > 28 days, and three reporting a duration of > 42 days. All three that reported a duration of > 42 days administered 8-week courses of C/T [56, 63, 67]. Two patients received C/T for XDR *P. aeruginosa* osteomyelitis [56, 67], and one received C/T for MDR *P. aeruginosa* mycotic pseudoaneurysm [63]. All patients had these infections following surgery.

### Outcomes

#### Overall outcomes

All 47 studies that included more than one patient reported clinical outcomes with C/T treatment: 39 reported clinical outcomes [18–21, 23–43, 81, 83–86, 90, 92, 94–100], 19 reported microbiological outcomes [19, 21, 24, 31–33, 35–38, 40, 41, 43, 80, 94, 96–98, 100], and 45 reported mortality rates [18–43, 79, 80, 82–92, 94–98, 100]. Clinical success rates ranged from 45.7 to 100.0%, with 27 studies (69%) reporting clinical success rates of > 70%. In larger studies (> 50 patients; 10 studies), clinical success rates ranged from 56.7 to 83.7%. Microbiological success rates were similar, ranging from 31 to 100%, with 14 studies (74%) reporting microbiological success rates of > 70%. In larger studies (> 50 patients; three studies), microbiological success rates ranged from 31 to 75.3%. Mortality rates ranged from 0 to 50%, with 31 studies (69%) reporting mortality rates of ≤ 20%. In larger studies (> 50 patients; 16 studies), mortality rates ranged from 5 to 29%. With each of these outcomes, note that definitions used, and assessments performed, were variable.

Outcomes were consistent in the 36 single-patient case reports—clinical cure was reported in 28 of 32 studies (87.5%), microbiological cure in 18 of 23 studies (78.3%), and mortality in 4 of 32 studies (11.4%).

#### Outcomes by treatment characteristics

Seven studies reported on the treatment characteristics that were risk factors for clinical outcomes [18, 25, 28, 30, 32, 33, 39]. Patient cohort size ranged from 21 to 205, with a median of 90. Five studies included patients with *P. aeruginosa* infections [25, 28, 30, 32, 33, 39]; one included patients with Enterobacterales infections [18]. There was a diverse range of infection types included.

Five studies found mixed evidence that a delay in receipt of C/T led to worse outcomes [18, 28, 30, 33, 39]. Bassetti et al. 2020 found that a significantly higher proportion of patients who achieved clinical success received empiric C/T and had a significantly shorter latency between infection onset and C/T administration (both p < 0.001) [18]. Similarly, Gallagher et al. 2018 found that starting C/T less than four days after positive culture was associated with significantly higher clinical and microbiological cure rates, and that starting C/T more than four days after positive culture was associated with significantly higher mortality [33]. In contrast, three studies found no association between initiating C/T within 48 h of *P. aeruginosa* isolation, time to C/T, or type of treatment (empiric, semi-empiric, or confirmed) (all p > 0.05) [28, 30, 39]. These three studies were of smaller size (169 combined patients vs. 258 for the two previously mentioned studies), and, importantly, Rodriguez-Nunez et al. included some patients that were also reported in Diaz-Cañestro et al*.* 2018, effectively double-counting these patients and possibly giving them disproportionate influence over the conclusion drawn in this review [28, 30].

#### Outcomes by pathogen

None of the publications identified conducted an analysis to determine the effect of pathogen type on outcomes. Bassetti et al. 2020 was the only large (> 50 patients) study to solely include patients with ESBL-producing Enterobacterales infections [18]. This study reported a clinical success of 83.7% and a mortality of 9.8% [18]. For descriptive comparison, there were 14 (1,632 total patients treated with C/T) comparably large (> 50 patients) studies that included patients with infections caused by *P. aeruginosa* [22, 25, 27, 28, 30, 33, 80, 83, 84, 88–91, 97]. Outcomes were comparable in these 14 studies, with clinical success ranging from 56.7 to 83.2% and mortality from 5 to 29%.

#### Outcomes by PsA resistance subtype

Two studies were identified that conducted an analysis to understand whether *P. aeruginosa* resistance was a factor in clinical outcome [28, 30]. In univariate analysis, Rodriguez-Nunez et al. found that similar proportions of survivors and non-survivors had XDR PsA infections [28]. Whereas, Diaz-Cañestro et al. found that resistance profile (the proportion of patients with MDR vs. XDR infections) was significantly different between patients who were clinical successes or failures (Table 3) [30].

**Table 3:** PsA resistance risk factors for clinical outcomes

Citation, study design, location	N C/T	Patient/infection description	Analysis	Variable	Proportion of patients with either outcome with variable		p-value
Rodriguez-Nunez et al. 2019 [28] Retrospective, multicenter International	90	Drug-resistant PsA RTIs (76.7% XDR; 23.3% MDR)	Univariate regression	XDR PsA infection	*Survivors* *(N* = *65)*	*Non-survivors* *(N* = *25)*	.308
					73.8% (48/65)	84.0% (21/25)	
Diaz-Cañestro et al*.* 2018 [30] Prospective, single center Spain	58	PsA (86.2% XDR; 10.3% MDR) infections, including RTIs (60.3%), UTIs (17.2%), and IAIs (6.9%)	Univariate regression		*Clinical* *cure* *(N* = *35)*	*Clinical failure (N* = *21)*	
				Resistance profile			**.045**
				XDR PsA infection	82.8% (29/35)	100.0% (21/21)	
				MDR PsA infection	17.1% (6/35)	0.0% (0/21)	

p-value < 0.05 indicates a significant difference are shown in bold

C/T: Ceftolozane/tazobactam; IAI: Intra-abdominal infection; MDR: Multidrug-resistant; PsA: *Pseudomonas aeruginosa*; RTI: Respiratory tract infection; UTI: Urinary tract infection; XDR: Extensively-drug-resistant

### Comparative studies

Five studies were identified that compared C/T with other treatment regimens (Table 4): three included aminoglycoside/polymyxin-based regimens as comparator [23, 27, 80]; two either used standard of care (SoC) [26, 83]. Each study included patients with *P. aeruginosa* infections, with four including patients with resistant *P. aeruginosa* [23, 27, 80, 83].

**Table 4:** Studies reporting comparative data

Citation, study design, location	Study design	Patient/infection description	Treatment groups	Outcome description	Outcome, % (n/N)		p-value/aOR
					C/T	Comparator	
*Aminoglycoside/polymyxin comparator*							
Caffrey et al. 2020 [80] Retrospective, multicenter, cohort US	Cohort	Patients had MDR PsA infections	C/T (N = 57) vs. aminoglycoside/polymyxin-based (N = 155)	Clinical cure	-	-	-
				Mortality, 30-day	17.5 (10/57)	18.1 (28/155)	aOR: 0.78 95% CI: 0.30–2.03
				Mortality, inpatient	15.8 (9/57)	27.7 (43/155)	**aOR: 0.39** **95% CI: 0.16–0.93**
				Microbiological cure	31.0 (13/42)	30.6 (33/108)	aOR: 0.88 95% CI: 0.35–2.21
Vena et al. 2020 [23] Retrospective, multicenter, case–control Italy	Case–control	Patients had pneumonia or bacteremia caused by MDR or XDR PsA	C/T (N = 16) vs. aminoglycoside/polymyxin-based (N = 32)	Clinical cure	81.3 (13/16)	56.3 (18/32)	0.11
				Mortality, 30-day	18.8 (3/16)	28.1 (9/32)	0.72
				Microbiological cure	-	-	-
Pogue et al. 2019 [27] Retrospective, multicenter, case–control US	Case–control	Patients had an MDR or XDR PsA infection	C/T (N = 100) vs. aminoglycoside/polymyxin-based (N = 100)	Clinical cure	81.0 (81/100)	61.0 (61/100)	**0.002**
				Mortality, in hospital	20.0 (20/100)	25.0 (25/100)	0.400
				Microbiological cure	-	-	-
*Other comparator*							
Fernández-Cruz et al. 2019 [26] Retrospective, single center, case–control Spain	Case–control	Patients had hematological malignancies and PsA infection	C/T (N = 19) vs. mixed SoC antibacterial agents (N = 38)	Clinical cure, 14-day	89.5 (17/19)	71.1 (27/38)	0.183
				Mortality, 30-day	5.3 (1/19)	28.9 (11/38)	**0.045**
				Microbiological cure	-	-	-
Mills et al*.* 2019 [83] Retrospective, multicenter cohort US	Cohort	Patients had pneumonia with an MDR PsA culture	C/T (N = 62) vs. mixed SoC antibacterial agents (N = 53)	Clinical cure, 14-day	72.6 (45/62)	67.9 (36/53)	0.683
				Mortality	29.0 (18/62)	26.4 (14/53)	0.840
				Microbiological cure	-	-	-

p-value < 0.05 indicates a significant difference are shown in bold

aOR: Adjusted odds ratio; CI: Confidence interval; C/T: Ceftolozane/tazobactam; IV: Intravenous; MDR: Multidrug-resistant; PsA: *Pseudomonas aeruginosa*; SoC: Standard of care; US: United States; XDR: Extensively-drug-resistant

In the three studies with aminoglycoside-/polymyxin-based comparators, all reported mortality rates [23, 27, 80], two reported clinical cure rates [23, 27], and one reported microbiological cure rate [80]. In Pogue et al., patients treated with C/T had significantly higher clinical cure rate (p = 0.002), but there was no difference in in-hospital mortality [27]. In response, Vena et al. conducted a similar case–control study, but balanced the proportion of patients with pneumonia in each arm, ensured patients received a sufficient polymyxin dosage, and ensured that all included patients had an infectious disease consultation [23]. Results were comparable with Pogue et al.—patients treated with C/T had a numerically higher clinical cure rate and lower mortality rate than patients treated with aminoglycoside/polymyxin regimen, though this did not reach statistical significance [23]. Caffrey et al. showed that patients treated with C/T were significantly less likely to die as inpatients than patients treated with aminoglycoside/polymyxin-based regimens, although there was no difference in 30-day mortality rates or microbiological cure rates, and clinical cure rates were not reported [80].

In the two studies that compared patients treated with C/T with mixed SoC antibacterial agents, both reported clinical cure rates and mortality [26, 83]. Both studies found that patients treated with C/T had numerically higher clinical cure rates than patients treated with other antibacterial agents. Fernández-Cruz et al*.* additionally found that patients treated with C/T had significantly lower mortality rates (p < 0.05) [26]; such a difference was not apparent in Mills et al. [83].

## Discussion

The principal finding of this SLR was that there is a body of RWE that establishes the effectiveness of C/T in real-world clinical practice, including patients described as severely ill patients and/or with resistant infections. Considering the patient disease severity measures, publications reported APACHE scores ranging from 13 to 40, with larger studies (> 50 patients) ranging from 18 to 40 [22, 24, 33, 36, 80, 84, 90, 97, 100]. This is higher than the APACHE score reported in ASPECT-NP (median 17) [15], and significantly higher than reported in ASPECT-IAI (mean 6.2) [16]. Furthermore, inclusion of immunocompromised patients, typically excluded by clinical trials, offers valuable insights into C/T effectiveness in this underrepresented population. A key limitation of many clinical trials is the exclusion of these seriously ill patients, and the restriction of recruitment to only patients with a narrow range of infections. By filling this gap, the RWE therefore provides valuable data on the outcomes of these patients seen in clinical practice.

Despite the heterogeneity in the patient population, outcomes of treatment with C/T were consistent with those found in the ASPECT clinical trials. In larger RWE studies (> 50 patients), clinical cure rates ranged from 56.7 to 83.7%, microbiological cure rates ranged from 31 to 75.3%, and mortality rates ranged from 5 to 29%. By way of descriptive comparison, C/T outcomes in the ASPECT trials were: ASPECT-cUTI, clinical cure = 92.0%, microbiological eradication = 80.4%, and mortality = 0.2% [17, 101]; ASPECT-cIAI, clinical cure = 83.0%, microbiological cure = 85.3%, and mortality = 2.3% [16, 102]; and ASPECT-NP, clinical cure = 54.4%, microbiological eradication = 73.1%, and 28-day mortality = 24.0% [15].

Treatment characteristics were broadly aligned with the approved use of C/T and both indicated doses of C/T were used approximately equally; however, it was unclear which dose was used for which indication and often the outcomes were not stratified by dose and indication. This result is concerning since the indicated dose for pneumonia is based on optimized pharmacokinetic and pharmacodynamic properties. C/T was more commonly used as confirmed therapy than as an empiric therapy (75.1% vs. 24.9%). This is consistent with the principles of antimicrobial stewardship, whereby broader-spectrum antibacterial agents are reserved for special clinical situations when other treatments have failed. However, there were two studies that suggested patients who were treated earlier; either empirically, or sooner after infection onset, had better clinical outcomes [18, 33], Although a similar association was not found in three other studies [28, 30, 39], comparison of early vs. late use of C/T warrants further investigation. Late use of C/T may be indicative of initial inappropriate antibacterial therapy with other agents, which has been shown in the literature to have deleterious effects on outcomes [8, 9].

Data from the comparative studies suggest that C/T is at least as effective as, and in several cases, significantly better than, aminoglycoside- or polymyxin-based regimens for serious, MDR infections [23, 27, 80]. Outside the scope of this review, though pertinent to clinicians, is the lower risk of nephrotoxicity with C/T compared to aminoglycosides or polymyxins. Both comparative studies that assessed safety found a significantly lower incidence of acute kidney injury with C/T than with aminoglycoside/polymyxin-based comparators [23, 27]. This combination of comparable effectiveness and lower risk of nephrotoxicity means that C/T can be an alternative to these therapies, particularly in patients with decreased renal function.

This SLR highlights the inconsistent reporting that is common within published RWE. Due to differences in study design, objectives, outcome assessment and definitions, there were often incomplete data for the variables of interest, as set out in this SLR. This variability in turn imposes challenges in attributing outcomes to the exposure studied. The inclusion of conference proceedings, which are not subject to the same rigorous peer-review, may have affected evidence included within this review, and thus the conclusions drawn. As mentioned in the results, some studies included data that were reported in part by other studies—this may be more widespread than thought as some large database studies collected patients across hundreds of hospitals, possibly capturing patients reported in other studies. As this is a qualitative review, this double counting was not adjusted for. However, given the consistency of outcomes between studies conducted in different locations, in different years, and by different authors, it is likely that the outcomes reported approximate the true treatment effect.

As was to be expected, many studies had small sample sizes and did not include comparison groups for statistical inference purposes. In the comparative cohort studies that did, C/T had comparable efficacy to standard of care, and was significantly better in several outcomes. Furthermore, identified risk factors may have been subject to a reporting bias: with some studies only reporting multivariate analysis, it was difficult to recognize which risk factors were non-significant, and therefore excluded, in univariate analysis. Moreover, the vast majority of publications were of a retrospective design. This may lead to selection bias, as both exposure and outcome of patients are already known. Many studies had industry authors and/or were sponsored by grants from industry which may lead to publication bias; however, the results appeared consistent regardless of authorship or sponsorship. Further publication bias may have arisen due to potential non-publication of negative results. Schumucker et al. found some evidence that meta-analyses which do not include unpublished or grey literature studies overestimate the treatment effect [103]. To mitigate this, this review included a search of recent ECCMID and IDWeek conference proceedings—two of the largest microbiology conferences in the US and Europe—to identify grey literature studies. However, this review did not include a comprehensive search of all relevant microbiology conferences or search for studies that were unpublished or preprints. Though these are pragmatic limitations associated with all literature reviews, there remains a possibility that the studies included in this review overestimate the treatment effect.

In conclusion, this SLR identified and summarized the published RWE on the use of C/T in clinical practice. These studies demonstrate the clinical effectiveness of C/T, despite the diverse group of seriously ill patients and level of resistance of the pathogens treated. The RWE body of literature provides additional insights into patient types that are commonly encountered in everyday practice and may have been excluded from the registration trials. Further studies are needed that evaluate homogenous patient sub-types and that account for other treatments that were received prior to C/T to properly attribute outcomes to the effectiveness of C/T.

## Supplementary Information

**Additional file 1:** PICOS criteria for study inclusion and Summary of single-patient case reports.

## Data Availability

All data analyzed during this study are included in this published article (and its supplementary information).

## Abbreviations

ABSSSI: Acute bacterial skin and skin structure infection
AIDS: Acquired immunodeficiency syndrome
aOR: Adjusted odds ratio
BSI: Bloodstream infection
CC(I): Charlson comorbidity (index)
CF: Cystic Fibrosis
CI: Confidence interval
cIAI: Complicated intra-abdominal infection
CLABSI: Central-line-associated bloodstream infection
CNS: Central nervous system
CR: Carbapenem-resistant
cSSTI: Complicated skin and soft tissue infection
C/T: Ceftolozane/tazobactam
cUTI: Complicated urinary tract infection
ECCMID: European Congress of Clinical Microbiology and Infectious Diseases
ESBL: Extended-spectrum β-lactamase
HABP: Hospital-acquired bacterial pneumonia
HAI: Hospital-acquired infection
HCAP: Healthcare-associated pneumonia
HIV: Human immunodeficiency syndrome
IAI: Intra-abdominal infection
ICU: Intensive care unit
IDWeek: Infectious Disease Week
IMC: Immunocompromised
IQR: Interquartile range
LOS: Length of stay
LVAD: Left-ventricular assist device
MDR: Multidrug-resistant
NP: Nosocomial pneumonia
NR: Not reported
OR: Odds ratio
PDR: Pandrug-resistant
PK/PD: Pharmacokinetics/pharmacodynamics
PsA: *Pseudomonas aeruginosa*
RCT: Randomized controlled trial
RTI: Respiratory tract infection
RWE: Real-world evidence
SD: Standard deviation
SLR: Systematic literature review
SoC: Standard of care
SOFA: Sequential Organ Failure Assessment
SSTI: Skin and soft tissue infection
UK: United Kingdom
US: United States
UTI: Urinary tract infection
VABP: Ventilator-associated bacterial pneumonia
WHO: World Health Organization
XDR: Extensively-drug-resistant

## References

[1] Centers for Disease Control and Prevention. Antibiotic resistant threats in the United States 2019. https://www.cdc.gov/drugresistance/pdf/threats-report/2019-ar-threats-report-508.pdf.

[2] Weiner LMWA, Limbago B (2016). Antimicrobial-resistant pathogens associated with healthcare-associated infections: summary of data reported to the National Healthcare Safety Network at the Centers for Disease Control and Prevention, 2011–2014. Infect Control Hosp Epidemiol.

[3] Peleg AYHD (2010). Hospital-acquired infections due to gram-negative bacteria. N Engl J Med.

[4] Ulu ACKB, Inal AS (2015). Risk factors of carbapenem-resistant *Klebsiella pneumoniae* infection: a serious threat in ICUs. Med Sci Monit: Int Med J Exp Clin Res.

[5] Infectious Diseases Society of America (2011). Combating antimicrobial resistance: policy recommendations to save lives. Clin Infect Dis.

[6] Martin-Loeches I, Torres A, Rinaudo M, Terraneo S, de Rosa F, Ramirez P (2015). Resistance patterns and outcomes in intensive care unit (ICU)-acquired pneumonia. Validation of European Centre for Disease Prevention and Control (ECDC) and the Centers for Disease Control and Prevention (CDC) classification of multidrug resistant organisms. J Infect.

[7] Tabak YP, Merchant S, Ye G, Vankeepuram L, Gupta V, Kurtz SG (2019). Incremental clinical and economic burden of suspected respiratory infections due to multi-drug-resistant *Pseudomonas aeruginosa* in the United States. J Hosp Infect.

[8] Bonine NG, Berger A, Altincatal A, Wang R, Bhagnani T, Gillard P (2019). Impact of delayed appropriate antibiotic therapy on patient outcomes by antibiotic resistance status from serious gram-negative bacterial infections. Am J Med Sci.

[9] Lodise TP, Zhao Q, Fahrbach K, Gillard PJ, Martin A (2018). A systematic review of the association between delayed appropriate therapy and mortality among patients hospitalized with infections due to *Klebsiella pneumoniae* or *Escherichia coli*: how long is too long?. BMC Infect Dis.

[10] Avent ML, Rogers BA, Cheng AC, Paterson DL (2011). Current use of aminoglycosides: indications, pharmacokinetics and monitoring for toxicity. Intern Med J.

[11] Ordooei Javan A, Shokouhi S, Sahraei Z (2015). A review on colistin nephrotoxicity. Eur J Clin Pharmacol.

[12] World Health Organization. Global priority list of antibiotic-resistant bacteria to guide research, discovery, and development of new antibiotics 2017. https://www.who.int/medicines/publications/WHO-PPL-Short_Summary_25Feb-ET_NM_WHO.pdf.

[13] Merck & Co. Ceftolozane/tazobactam (ZERBAXA®) [Prescribing information]. 2019.

[14] Merck Sharp & Dohme. Ceftolozane/tazobactam (Zerbaxa) Summary of Product Characteristics. 2019.

[15] Kollef MH, Nováček M, Kivistik Ü, Réa-Neto Á, Shime N, Martin-Loeches I (2019). Ceftolozane-tazobactam versus meropenem for treatment of nosocomial pneumonia (ASPECT-NP): a randomised, controlled, double-blind, phase 3, non-inferiority trial. Lancet Infect Dis.

[16] Solomkin J, Hershberger E, Miller B, Popejoy M, Friedland I, Steenbergen J (2015). Ceftolozane/tazobactam plus metronidazole for complicated intra-abdominal infections in an era of multidrug resistance: results from a randomized, double-blind, phase 3 trial (ASPECT-cIAI). Clin Infect Dis.

[17] Wagenlehner FM, Umeh O, Steenbergen J, Yuan G, Darouiche RO (2015). Ceftolozane-tazobactam compared with levofloxacin in the treatment of complicated urinary-tract infections, including pyelonephritis: a randomised, double-blind, phase 3 trial (ASPECT-cUTI). The Lancet.

[18] Bassetti M, Vena A, Giacobbe DR, Falcone M, Tiseo G, Giannella M (2020). Ceftolozane/tazobactam for treatment of severe ESBL-producing enterobacterales infections: a multicenter nationwide clinical experience (CEFTABUSE II Study). Open Forum Infect Dis.

[19] Bosaeed M, Ahmad A, Alali A, Mahmoud E, Alswidan L, Alsaedy A (2020). Experience with ceftolozane-tazobactam for the treatment of serious *Pseudomonas aeruginosa* infections in Saudi Tertiary Care Center. Infect Dis: Res Treat.

[20] Buonomo AR, Maraolo AE, Scotto R, Foggia M, Zappulo E, Congera P (2020). Efficacy and safety of ceftolozane/tazobactam as therapeutic option for complicated skin and soft tissue infections by MDR/XDR *Pseudomonas aeruginosa* in patients with impaired renal function: a case series from a single-center experience. Infection.

[21] Jones BM, Huelfer K, Bland CM (2020). Clinical and safety evaluation of continuously infused ceftolozane/ tazobactam in the outpatient setting. Open Forum Infect Dis.

[22] Jorgensen SCJ, Trinh TD, Zasowski EJ, Lagnf AM, Simon SP, Bhatia S (2020). Real-world experience with ceftolozane-tazobactam for multidrug-resistant gram-negative bacterial infections. Antimicrob Agents Chemother.

[23] Vena A, Giacobbe DR, Mussini C, Cattelan A, Bassetti M, Ceftabuse Study Group (2020). Clinical efficacy of ceftolozane-tazobactam versus other active agents for the treatment of bacteremia and nosocomial pneumonia due to drug-resistant *Pseudomonas aeruginosa*. Clin Infect Dis.

[24] Gerlach AT, Goff DA, Bazan JA (2019). Ceftolozane/tazobactam for the treatment of osteomyelitis due to multidrug-resistant *Pseudomonas aeruginosa*. Infect Dis Clin Pract.

[25] Bassetti M, Castaldo N, Cattelan A, Mussini C, Righi E, Tascini C (2019). Ceftolozane/tazobactam for the treatment of serious *Pseudomonas aeruginosa* infections: a multicentre nationwide clinical experience. Int J Antimicrob Agents.

[26] Fernandez-Cruz A, Alba N, Semiglia-Chong MA, Padilla B, Rodriguez-Macias G, Kwon M (2019). A case-control study of real-life experience with ceftolozane-tazobactam in patients with hematologic malignancy and pseudomonas aeruginosa infection. Antimicrob Agents Chemother.

[27] Pogue JM, Kaye KS, Veve MP, Patel TS, Gerlach AT, Davis SL (2019). Ceftolozane/tazobactam vs polymyxin or aminoglycoside-based regimens for the treatment of drug-resistant *Pseudomonas aeruginosa*. Clin Infect Dis: Off Publ Infect Dis Soc Am.

[28] Rodriguez-Nunez O, Perianez-Parraga L, Oliver A, Munita JM, Bote A, Gasch O (2019). Higher MICs (>2 mg/L) predict 30-day mortality in patients with lower respiratory tract infections caused by multidrug- and extensively drug-resistant *Pseudomonas aeruginosa* treated with ceftolozane/tazobactam. Open Forum Infect Dis.

[29] Tan X, Moenster RP (2019). Ceftolozane-tazobactam for the treatment of osteomyelitis caused by multidrug-resistant pathogens: a case series. Ther Adv Drug Saf.

[30] Diaz-Canestro M, Perianez L, Mulet X, Martin-Pena ML, Fraile-Ribot PA, Ayestaran I (2018). Ceftolozane/tazobactam for the treatment of multidrug resistant *Pseudomonas aeruginosa*: experience from the Balearic Islands. Eur J Clin Microbiol Infect Dis.

[31] Dietl B, Sanchez I, Arcenillas P, Cuchi E, Gomez L, de Molina FJG (2018). Ceftolozane/tazobactam in the treatment of osteomyelitis and skin and soft-tissue infections due to extensively drug-resistant *Pseudomonas aeruginosa*: clinical and microbiological outcomes. Int J Antimicrob Agents.

[32] Escola-Verge L, Pigrau C, Los-Arcos I, Arevalo A, Vinado B, Campany D (2018). Ceftolozane/tazobactam for the treatment of XDR *Pseudomonas aeruginosa* infections. Infection.

[33] Gallagher JC, Satlin MJ, Elabor A, Saraiya N, McCreary EK, Molnar E (2018). Ceftolozane-tazobactam for the treatment of multidrug-resistant *Pseudomonas aeruginosa* Infections: a multicenter study. Open Forum Infect Dis.

[34] Hakki M, Lewis JS (2018). Ceftolozane-tazobactam therapy for multidrug-resistant *Pseudomonas aeruginosa* infections in patients with hematologic malignancies and hematopoietic-cell transplant recipients. Infection.

[35] Xipell M, Paredes S, Fresco L, Bodro M, Marco F, Martinez JA (2018). Clinical experience with ceftolozane/tazobactam in patients with serious infections due to resistant *Pseudomonas aeruginosa*. J Global Antimicrob Resist.

[36] Alvarez Lerma F, Munoz Bermudez R, Grau S, Gracia Arnillas MP, Sorli L, Recasens L (2017). Ceftolozane-tazobactam for the treatment of ventilator-associated infections by colistin-resistant *Pseudomonas aeruginosa*. Revista Espanola de Quimioterapia.

[37] Caston JJ, De La Torre A, Ruiz-Camps I, Sorli ML, Torres V, Torre-Cisneros J (2017). Salvage therapy with ceftolozane-tazobactam for multidrug-resistant *Pseudomonas aeruginosa* infections. Antimicrob Agents Chemother..

[38] Dinh A, Wyplosz B, Kerneis S, Lebeaux D, Bouchand F, Duran C (2017). Use of ceftolozane/tazobactam as salvage therapy for infections due to extensively drug-resistant *Pseudomonas aeruginosa*. Int J Antimicrob Agents.

[39] Haidar G, Philips NJ, Shields RK, Snyder D, Cheng S, Potoski BA (2017). Ceftolozane-tazobactam for the treatment of multidrug-resistant *Pseudomonas aeruginosa* infections: clinical effectiveness and evolution of resistance. Clin Infect Dis.

[40] Munita JM, Aitken SL, Miller WR, Perez F, Rosa R, Shimose LA (2017). Multicenter evaluation of ceftolozane/tazobactam for serious infections caused by carbapenem-resistant *Pseudomonas aeruginosa*. Clin Infect Dis.

[41] Sacha GL, Neuner EA, Athans V, Bass SN, Pallotta A, Rivard KR (2017). Retrospective evaluation of the use of ceftolozane/tazobactam at a large academic medical center. Infect Dis Clin Pract.

[42] Xipell M, Bodro M, Marco F, Martinez JA, Soriano A (2017). Successful treatment of three severe MDR or XDR *Pseudomonas aeruginosa* infections with ceftolozane/tazobactam. Future Microbiol.

[43] Gelfand MS, Cleveland KO (2015). Ceftolozane/tazobactam therapy of respiratory infections due to multidrug-resistant pseudomonas aeruginosa. Clin Infect Dis.

[44] Mahmoud A, Shah A, Nutley K, Nicolau DP, Sutherland C, Jain M (2020). Clinical pharmacokinetics of ceftolozane and tazobactam in an obese patient receiving continuous venovenous haemodiafiltration: a patient case and literature review. J Global Antimicrob Resist.

[45] Romano MT, Premraj S, Bray JM, Murillo LC (2020). Ceftolozane/tazobactam for pulmonary exacerbation in a 63-year-old cystic fibrosis patient with renal insufficiency and an elevated MIC to *Pseudomonas aeruginosa*. IDCases.

[46] Maddocks S, Fabijan AP, Ho J, Lin RCY, Ben Zakour NL, Dugan C (2019). Bacteriophage therapy of ventilator-associated pneumonia and empyema caused by pseudomonas aeruginosa. Am J Respir Crit Care Med.

[47] Aguilar G, Ferriols R, Martinez-Castro S, Ezquer C, Pastor E, Carbonell JA (2019). Optimizing ceftolozane-tazobactam dosage in critically ill patients during continuous venovenous hemodiafiltration. Crit Care.

[48] Arena F, De Angelis LH, Maglioni E, Contorni M, Cassetta MI, Novelli A (2019). Ceftolozane-tazobactam pharmacokinetics during extracorporeal membrane oxygenation in a lung transplant recipient. Antimicrob Agents Chemother.

[49] Carbonell N, Aguilar G, Ferriols R, Huerta R, Ferreres J, Calabuig M (2019). Ceftolozane pharmacokinetics in a septic critically ill patient under different extracorporeal replacement therapies. Antimicrob Agents Chemother.

[50] Davis SE, Ham J, Hucks J, Gould A, Foster R, Ann Justo J (2019). Use of continuous infusion ceftolozane-tazobactam with therapeutic drug monitoring in a patient with cystic fibrosis. Am J Health Syst Pharm.

[51] Gonzales Zamora JA, Varadarajalu Y (2019). Fatal Curvularia brain abscess in a heart and kidney transplant recipient. IDCases.

[52] Pezzi M, Scozzafava AM, Giglio AM, Vozzo R, Casella PD, Tiburzi SP (2019). Use of ceftolozane/tazobactam in a case of septic shock by puerperal sepsis. Case Rep Obstet Gynecol.

[53] Saraca LM, Di Giuli C, Sicari F, Priante G, Lavagna F, Francisci D (2019). Use of ceftolozane-tazobactam in patient with severe medium chronic purulent otitis by XDR *Pseudomonas aeruginosa*. Case Rep.

[54] Alessa MA, Almangour TA, Alhossan A, Alkholief MA, Alhokail M, Tabb DE (2018). Ceftolozane-tazobactam for the treatment of multidrug-resistant *Pseudomonas aeruginosa* pneumonia in a patient receiving intermittent hemodialysis. Am J Health Syst Pharm.

[55] Frattari A, Savini V, Polilli E, Cibelli D, Talamazzi S, Bosco D (2018). Ceftolozane-tazobactam and fosfomycin for rescue treatment of otogenous meningitis caused by XDR *Pseudomonas aeruginosa*: case report and review of the literature. IDCases.

[56] Hassan S, Kahn MD, Saraiya N, Nori P (2018). Treatment of a complex orthopaedic infection due to extensively drug-resistant *Pseudomonas aeruginosa*. BMJ Case Rep.

[57] Lewis PO, Cluck DB, Tharp JL, Krolikowski MA, Patel PD (2018). Failure of ceftolozane-tazobactam salvage therapy in complicated pneumonia with lung abscess. Clin Case Rep.

[58] Monterrubio-Villar J, Rodriguez-Garrido S, Jimenez-Delgado JD (2018). Postoperative soft-tissue infection due to multidrug-resistant *Pseudomonas aeruginosa*: usefulness of ceftolozane-tazobactam. Revista Espanola de Quimioterapia.

[59] So W, Shurko J, Galega R, Quilitz R, Greene JN, Lee GC (2019). Mechanisms of high-level ceftolozane/tazobactam resistance in *Pseudomonas aeruginosa* from a severely neutropenic patient and treatment success from synergy with tobramycin. J Antimicrob Chemother.

[60] Stewart A, Roberts JA, Wallis SC, Allworth AM, Legg A, McCarthy KL (2018). Evidence of clinical response and stability of ceftolozane/tazobactam used to treat a carbapenem-resistant *Pseudomonas aeruginosa* lung abscess on an outpatient antimicrobial program. Int J Antimicrob Agents.

[61] Stokem K, Zuckerman JB, Nicolau DP, Wungwattana M, Sears EH (2018). Use of ceftolozane-tazobactam in a cystic fibrosis patient with multidrug-resistant pseudomonas infection and renal insufficiency. Respir Med Case Rep.

[62] Teleb M, Soto-Ruiz E, Dominguez DC, Antony S (2018). ESBL *E. coli* and *P. aeruginosa* resistance to ceftolozane-tazobactam in a patient with a liver abscess. The search for an omnipotent antibiotic goes on. Infect Disord - Drug Targets.

[63] Aye C, Williams M, Horvath R (2017). Multidrug resistant pseudomonas mycotic pseudoaneurysm following cardiac transplant bridged by ventricular assistant device. Case rep.

[64] Castaldo N, Givone F, Peghin M, Righi E, Sartor A, Bassetti M (2017). Multidrug-resistant *Pseudomonas aeruginosa* skin and soft-tissue infection successfully treated with ceftolozane/tazobactam. J Global Antimicrob Resist.

[65] Dinh A, Davido B, Calin R, Paquereau J, Duran C, Bouchand F (2017). Ceftolozane/tazobactam for febrile UTI due to multidrug-resistant *Pseudomonas aeruginosa* in a patient with neurogenic bladder. Spinal Cord Ser Cases.

[66] Dominguez AS, Perez-Rodriguez MT, Nodar A, Martinez-Lamas L, Perez-Landeiro A, Casal MC (2017). Successful treatment of MDR *Pseudomonas aeruginosa* skin and soft-tissue infection with ceftolozane/tazobactam. J Antimicrob Chemother.

[67] Gentile I, Buonomo AR, Maraolo AE, Scotto R, De Zottis F, Di Renzo G (2017). Successful treatment of post-surgical osteomyelitis caused by XDR *Pseudomonas aeruginosa* with ceftolozane/tazobactam monotherapy. J Antimicrob Chemother.

[68] Hernandez-Tejedor A, Merino-Vega CD, Martin-Vivas A, de Luna-Gonzalez RR, Delgado-Iribarren A, Gaban-Diez A (2017). Successful treatment of multidrug-resistant *Pseudomonas aeruginosa* breakthrough bacteremia with ceftolozane/tazobactam. Infection.

[69] Jones BM, Smith B, Bland CM (2017). Use of continuous-infusion ceftolozane/tazobactam in a multidrug-resistant *Pseudomonas aeruginosa* urinary tract infection in the outpatient setting. Ann Pharmacother.

[70] Kurtzhalts KE, Mergenhagen KA, Manohar A, Berenson CS (2017). Successful treatment of multidrug-resistant *Pseudomonas aeruginosa* pubic symphysis osteomyelitis with ceftolozane/tazobactam. BMJ Case Re.

[71] MacVane SH, Pandey R, Steed LL, Kreiswirth BN, Chen L (2017). Emergence of ceftolozane-tazobactam-resistant *Pseudomonas aeruginosa* during treatment is mediated by a single AmpC structural mutation. Antimicrob Agents Chemother.

[72] Peghin M, Maiani M, Castaldo N, Givone F, Righi E, Lechiancole A (2018). Ceftolozane/tazobactam for the treatment of MDR *Pseudomonas aeruginosa* left ventricular assist device infection as a bridge to heart transplant. Infection.

[73] Schwarz ER, Oikonomou KG, Reynolds M, Kim J, Balmiki RL, Sterling SA (2017). Extranodal NK/T-cell lymphoma, nasal type, presenting as refractory pseudomonas aeruginosa facial cellulitis. J Investig Med High Impact Case Rep.

[74] Jolliff JC, Ho J, Joson J, Heidari A, Johnson R (2016). Treatment of polymicrobial osteomyelitis with ceftolozane-tazobactam: case report and sensitivity testing of isolates. Case Rep.

[75] Kuti JL, Ghazi IM, Quintiliani R, Shore E, Nicolau DP (2016). Treatment of multidrug-resistant *Pseudomonas aeruginosa* with ceftolozane/tazobactam in a critically ill patient receiving continuous venovenous haemodiafiltration. Int J Antimicrob Agents.

[76] Patel UC, Nicolau DP, Sabzwari RK (2016). Successful treatment of multi-drug resistant *Pseudomonas aeruginosa* bacteremia with the recommended renally adjusted ceftolozane/tazobactam regimen. Infect Dis Therapy.

[77] Vickery SB, McClain D, Wargo KA (2016). Successful use of ceftolozane-tazobactam to treat a pulmonary exacerbation of cystic fibrosis caused by multidrug-resistant *Pseudomonas aeruginosa*. Pharmacotherapy.

[78] Soliman R, Lynch S, Meader E, Pike R, Turton JF, Hill RL (2015). Successful ceftolozane/tazobactam treatment of chronic pulmonary infection with pan-resistant *Pseudomonas aeruginosa*. JMM Case Rep.

[79] Gudiol A, Fernandez-Cruz P, Hakki R (2020). Ceftolozane-tazobactam for the treatment of bloodstream infection due to *Pseudomonas aeruginosa* in neutropenic cancer patients: a real-life experience (ZENITH study).

[80] Caffrey P, Lopes P, Laplante (2020). Comparative effectiveness of ceftolozane/tazobactam versus aminoglycosides or polymyxins in multidrug resistant *Pseudomonas aeruginosa* infections.

[81] Trisler MJ, Tamma P, Avdic E (2019). Comparison of outcomes between patients with and without cystic fibrosis treated with ceftolozane-tazobactam for *Pseudomonas aeruginosa* infections.

[82] Sheffield N, O'Neal G, Bouchard N (2019). The use of continuous infusion ceftolozane/tazobactam for resistant gram-negative bacterial infections: a case series.

[83] Mills, MacWhinnie, Do, editors. Evaluating the impact of ceftolozane/tazobactam on clinical outcomes in patients with multi-drug-resistant *Pseudomonas aeruginosa* pneumonia. San Diego, CA: IDWeek; 2019.

[84] Hart G (2019). Ceftolozane–tazobactam (C/T) treatment outcomes in immunocompromised (IC) patients with multidrug-resistant (MDR) *Pseudomonas aeruginosa* (PA) infections.

[85] Cabrera T, Miller D, Hanson M (2019). Clinical and microbiological outcomes associated with real-world use of ceftolozane/tazobactam.

[86] Tordato C, Lagioia M (2018). Efficacy and safety of ceftolozane/tazobactam as salvage therapy in severely ill patients.

[87] Puzniak L, Fu R, Gundrum J (2018). Real world evaluation of patient characteristics and outcomes of patients treated with ceftolozane/tazobactam across 253 US hospitals.

[88] Puzniak L, Fu R, Gundrum J, eds. Real world evaluation of ceftolozane/tazobactam treatment for Pseudomonas across 253 US hospitals. SCCM; 2018.

[89] Pogue J, Sanagaram R, Merchant S, editors. Real world clinical experience with ceftolozane/tazobactam (C/T) for the treatment of complicated urinary tract infections (cUTI) and complicated intraabdominal infections (cIAI) due to *Pseudomonas aeruginosa* (PSA): an electronic medical record database review in the United States. Munich: ECCMID; 2018.

[90] Jorgensen T, Lagnf B, Estrada S (2018). Multicentre evaluation of ceftolozane-tazobactam for multidrug-resistant *Pseudomonas aeruginosa* infections.

[91] Jorgensen et al., eds. Multicenter evaluation of C/T monotherapy vs. combination therapy for MDR *P. aeruginosa*. ASM Microbe; 2018.

[92] Jayakumar L, Kullar H, Nguyen P (2018). Real-world evaluation of ceftolozane/tazobactam (C/T) in severely ill patients with sepsis and/or bacteraemia.

[93] Hooper C, Elsayed B, Bailey B, editors. Successful treatment of chronic spinal osteomyelitis caused by multidrug resistant *Pseudomonas aeruginosa* with ceftolozane-tazobactam and surgical intervention. Can J Hosp Pharmacy. 2018;71(1):58–9.

[94] Hirsch H, Piche C, Beaulac B, et al., eds. A multi-center evaluation of outcomes following treatment with ceftolozane-tazobactam; 2018.

[95] Henry S, Puzniak R, Van Schooneveld B (2018). Ceftolozane-tazobactam use and outcomes at an academic transplant center.

[96] Gioia S, Martín-Dávila P, Reilly R-G (2018). Ceftolozane-tazobactam for the treatment of *Pseudomonas aeruginosa* infection in a tertiary hospital: clinical outcome and develop of resistance.

[97] Elabor M, King G (2018). Ceftolozane/tazobactam for the treatment of multidrug resistant *Pseudomonas aeruginosa* infections in immunocompromised patients: a multi center study.

[98] Leuthner K, Jayakumar H, Nguyen P (2017). Real-world evaluation of ceftolozane/tazobactam (C/T) use and clinical outcomes at an Academic Medical Center in Las Vegas.

[99] Nathan A, Prokesch Q, Sleweon S (2016). Ceftolozane/tazobactam: outpatient treatment of gram-negative infections at Physician Office Infusion Centers (POICs).

[100] Iovleva M, Perez R, Jacobs B (2016). Ceftazidime/avibactam and ceftolozane/tazobactam in treatment of pulmonary infections by Imipenem resistant *Pseudomonas aeruginosa*.

[101] Merck & Co. Data on file: ASPECT-cUTI Clinical Study Report. 2014.

[102] Merck & Co. Data on file: ASPECT-cIAI Clinical Study Report. 2014.

[103] Schmucker CM, Blümle A, Schell LK, Schwarzer G, Oeller P, Cabrera L (2017). Systematic review finds that study data not published in full text articles have unclear impact on meta-analyses results in medical research. PLoS ONE.

