# Supplementary material for: Real-world use of ceftolozane/tazobactam: a systematic literature review
Source: Antimicrob Resist Infect Control. 2021 Apr 8;10:68. doi: 10.1186/s13756-021-00933-8 (PMC8027296; doi:10.1186/s13756-021-00933-8)
Supplement: Supplementary file 1 — Additional file 1: PICOS criteria for study inclusion and Summary of single-patient case reports. [file 13756_2021_933_MOESM1_ESM.docx]

**Supplementary material**

**PICOS criteria**

**Table S1. PICOS criteria for study inclusion**

| **PICOS** | **Inclusion criteria** | **Exclusion criteria** |
| --- | --- | --- |
| Population | Adult patients (≥18 years) with gram-negative infections | Studies not reporting data on adult patients with gram-negative infections |
| Interventions | Ceftolozane/tazobactam | Publications which report solely on interventions other than ceftolozane/tazobactam |
| Comparator | N/A | N/A |
| Outcomes | Any relevant efficacy (e.g. clinical, microbiological, mortality-based) outcome | Any outcome not in the inclusion criteria (e.g. PK/PD, susceptibility, economic outcomes) |
| Study design | Non-randomized and non-controlled studies from real-world clinical practice (e.g. observational studies, retrospective studies, case-control studies) | Randomized or controlled studies (e.g. randomized controlled trials)  Non-clinical studies (e.g. *in vitro*)  Commentaries, editorials, literature reviews or letters without data presented |

N/A: Not applicable; PK/PD: Pharmacokinetics/pharmacodynamics.

**Case report summaries**

**Table S2. Summary of single-patient case reports**

| **Citation, location** | **N** | **Patient/infection description** | **Disease severity** | **C/T treatment** | **Outcome, %** | | |
| --- | --- | --- | --- | --- | --- | --- | --- |
|  |  |  |  |  | **Clinical** | **Micro.** | **Mortality** |
| **2020 studies** |  |  |  |  |  |  |  |
| **Mahmoud** *et al*. 2020(44)  US | 1 | MDR PsA RTI. | ICU N=1 | Dose C/T: 3 g q8h initially, then 9 g qd CI  Empiric/confirmed C/T: Confirmed  Duration: 7 days | - | 100 | - |
| **Romano** *et al*. 2020(45)  US | 1 | MDR PsA pulmonary exacerbation of cystic fibrosis. | - | Dose C/T: 3 g q8h  Empiric/confirmed C/T: Confirmed  Duration: 14 days | 100 | - | 0 |
| **2019 studies** |  |  |  |  |  |  |  |
| **Aguilar** *et al.* 2019(47)  Spain | 1 | PsA nosocomial peritonitis and septic shock. | ICU N=1 | Dose C/T: 3 g q8h | 100 | 100 | 0 |
| **Arena** *et al.* 2019(48)  Italy | 1 | CR PsA pulmonary infection in a lung transplant recipient. | IMC N=1 | Dose C/T: 3 g q8h  Empiric/confirmed C/T: Confirmed  Duration: 15 days | 100 | 100 | 0 |
| **Carbonell** *et al*. 2019(49)  Spain | 1 | PsA septic shock secondary to catheter-related bacteremia. | ICU N=1  IMC N=1 | Dose C/T: 3 g q3h  Empiric/confirmed C/T: Empiric | 0 | 0 | 100 |
| **Davis** *et al*. 2019(50)  US | 1 | MDR PsA and ESBL-producing *E. coli* pulmonary exacerbation of cystic fibrosis. | ICU N=1 | Dose C/T: 6 g CI qd  Empiric/confirmed C/T: Confirmed | 100 | - | 0 |
| **Gonzales Zamora** *et al*. 2019(51)  US | 1 | MDR PsA and *Curvularia* spp. (a species of fungus) pneumonia and bacteremia, then *Curvularia* spp. brain abscess. | IMC N=1 | Duration: 14 days | - | - | 100^a^ |
| **Maddocks** *et al.* 2019(46)  Australia | 1 | PsA VABP. | ICU N=1 | Dose C/T: 1.5 g q8h  Empiric/confirmed C/T: Confirmed, as desensitization therapy  Duration: 42 days | 100^b^ | 100^b^ | 0^b^ |
| **Pezzi** *et al.* 2019(52)  Italy | 1 | ESBL-producing *Klebsiella* spp. and MDR *E. coli* septic shock by puerperal sepsis. | ICU N=1 | Dose C/T: 1.5 g q8h  Empiric/confirmed C/T: Confirmed  Duration: 14 days | 100 | 100 | 0 |
| **Saraca** *et al*. 2019(53)  Italy | 1 | XDR PsA chronic purulent otitis. | IMC N=1 | Dose C/T: 3 g q8h  Empiric/confirmed C/T: Confirmed  Duration: 16/17 days^c^ | 100 | 100 | 0 |
| **So** *et al.* 2018(59)  US | 1 | MDR PsA bacteremia in a severely neutropenic patient. | IMC N=1 | Dose C/T: 3 g q8h  Empiric/confirmed C/T: Confirmed  Duration: 37 days | 100^d^ | - | 0^d^ |
| **2018 studies** |  |  |  |  |  |  |  |
| **Alessa** *et al.* 2018(54)  US | 1 | MDR PsA NP in a patient receiving hemodialysis. | - | Dose C/T: 1.5 g loading dose then 0.3 g q8h  Empiric/confirmed C/T: Confirmed  Duration: 13 days | 100 | 100 | 0 |
| **Frattari** *et al.* 2018(55)  Italy | 1 | XDR PsA otogenous meningitis. | ICU N=1 | Dose C/T: 3 g q8h  Empiric/confirmed C/T: Confirmed  Duration: 14 days | 100 | 100 | 0 |
| **Hassan** *et al.* 2018(56)  US | 1 | XDR PsA osteomyelitis. | ICU N=0 | Dose C/T: 1.5 g q8h  Empiric/confirmed C/T: Confirmed  Duration: 8 weeks | 100 | 100 | 0 |
| **Hooper** *et al*. 2018(93)  Canada | 1 | MDR PsA and *S. anginosus c*hronic spinal osteomyelitis. | - | Dose C/T: 3 g q8h  Empiric/confirmed C/T: Confirmed  Duration: 6 weeks | 100 | 100 | 0 |
| **Lewis** *et al.* 2018(57)  US | 1 | MDR PsA HCAP. | - | Dose C/T: 1.5 g q8h  Empiric/confirmed C/T: Empiric  Duration: 12 days | 0 | 0 | 100 |
| **Monterrubio-Villar** *et al.* 2018(58)  Spain | 1 | MDR PsA SSTI. | ICU N=1 | Dose C/T: 0.75 g q8h  Empiric/confirmed C/T: Confirmed  Duration: 7 days | 100 | 100 | 0 |
| **Peghin** *et al.* 2018(72)  Italy | 1 | MDR PsA LVAD related infection. | - | Dose C/T: 1.5 g q8h  Empiric/confirmed C/T: Confirmed  Duration: 6 weeks / 4.5 weeks^e^ | 100^e^ | 100^e^ | 0^e^ |
| **Stewart** *et al.* 2018(60)  Australia | 1 | MDR PsA pulmonary infection in kidney transplant. | ICU N=1  IMC N=1 | Dose C/T: 4.5 g qd continuous infusion  Empiric/confirmed C/T: Confirmed  Duration: 42 days | 100 | - | 0 |
| **Stokem** *et al.* 2018(61)  US | 1 | MDR PsA pulmonary exacerbation of cystic fibrosis. | IMC N=1 | Dose C/T: 3 g q12h  Duration: 14 days | 100 | - | 0 |
| **Teleb** *et al.* 2018(62)  US | 1 | MDR PsA and ESBL-producing *E. coli l*iver abscess. | - | Empiric/confirmed C/T: Empiric | 0 | 0 | 100 |
| **2017 studies** |  |  |  |  |  |  |  |
| **Aye** *et al.* 2017(63)  Australia | 1 | MDR PsA mycotic pseudoaneurysm in a patient following heart transplant. | IMC N=1 | Dose C/T: 1.5 g q8h  Empiric/confirmed C/T: Empiric  Duration: 8 weeks | 100 | 100 | 0 |
| **Castaldo** *et al.* 2017(64)  Italy | 1 | MDR PsA SSTI. | - | Dose C/T: 1.5 g q8h  Empiric/confirmed C/T: Confirmed  Duration: 14 days | 100 | - | 0 |
| **Dinh** *et al.* 2017(65)  France | 1 | MDR PsA febrile UTI. | - | Dose C/T: 1.5 g q8h  Empiric/confirmed C/T: Confirmed  Duration: 7 days | 100 | 100 | 0 |
| **Dominguez** *et al.* 2017(66)  Spain | 1 | MDR PsA cSSTI. | - | Dose C/T: 0.375 g q8h then 1.5 g q8h  Empiric/confirmed C/T: Empiric  Duration: 14 days | 100 | - | 0 |
| **Gentile** *et al.* 2017(67)  Italy | 1 | XDR PsA osteomyelitis. | - | Dose C/T: 0.75 g q8h then 1.5 g q8h  Empiric/confirmed C/T: Empiric  Duration: 8 weeks | 100 | - | 0 |
| **Hernández-Tejedor** *et al.* 2017(68)  US | 1 | MDR PsA ventilator-associated tracheobronchitis. | ICU N=1  IMC N=1 | Dose C/T: 1.5 g q8h  Empiric/confirmed C/T: Confirmed  Duration: 10 days | 100 | 100 | 0 |
| **Jones** *et al.* 2017(69)  US | 1 | MDR PsA UTI. | - | Dose C/T: 4.5 g/24h CI  Empiric/confirmed C/T: Empiric  Duration: 2 weeks | 100 | 100 | 0 |
| **Kurtzhalts** *et al.* 2017(70)  US | 1 | MDR PsA osteomyelitis. | - | Dose C/T: 1.5 g q8h  Empiric/confirmed C/T: Confirmed  Duration: 6 weeks | 100 | 100 | 0 |
| **MacVane** *et al.* 2017(71)  US | 1 | MDR PsA wound infection. | - | Dose C/T: 1.5 g q8h  Empiric/confirmed C/T: Confirmed  Duration: 6 weeks | 0^f^ | 0^f^ | 100^f^ |
| **Schwarz** *et al.* 2017(73)  US | 1 | XDR PsA facial cellulitis and extranodal natural killer T-cell lymphoma, septic shock. | ICU N=1  IMC N=1 | Dose C/T: 1.5 g q8h | 100 | - | 0 |
| **2016 studies** |  |  |  |  |  |  |  |
| **Jolliff** *et al.* 2016(74)  US | 1 | MDR *S. maltophilia* polymicrobial osteomyelitis. | - | Dose C/T: 1.5 g q8h  Empiric/confirmed C/T: Confirmed  Duration: 6 weeks | 100 | 100 | 0 |
| **Kuti** *et al.* 2016(75)  US | 1 | MDR PsA VABP. | ICU N=1 | Dose C/T: 3 g q8h  Empiric/confirmed C/T: Confirmed  Duration: 10 days | 100 | 100 | 0 |
| **Patel** *et al.* 2016(76)  US | 1 | MDR PsA, *P. mirabilis*, and *K. pneumoniae* BSI. | - | Dose C/T: 0.375 q8h  Empiric/confirmed C/T: Confirmed  Duration: 25 days | 100 | 100 | 0 |
| **Vickery** *et al.* 2016(77)  US | 1 | MDR PsA pulmonary exacerbation of cystic fibrosis. | - | Dose C/T: 3 g q8h  Empiric/confirmed C/T: Confirmed  Duration: 12 days | 100 | - | 0 |
| **Soliman** *et al.* 2015(78)  UK | 1 | PDR PsA exacerbation of chronic pulmonary infection (bronchiectasis). | - | Dose C/T: 3 g q8h  Empiric/confirmed C/T: Confirmed  Duration: 14 days | 100 | 100 | 0 |

^a^The patient died of multiorgan failure, it is unclear from the publication whether this was due to the PsA infection or the *Curvularia* spp. brain abscess.

^b^Patient was started on C/T 5 days after starting bacteriophage therapy. The publication notes that ‘at this time the patient had made “remarkable progress over the last week”’ (after starting bacteriophage therapy).

^c^Therapy described as “*From 17th October to 2nd November, the day of discharge, the patient underwent antibiotic therapy with C/T..*.”. Including the day of discharge, C/T duration was 17 days; excluding the day of discharge, C/T duration was 16 days.

^d^Susceptibility tests showed that the isolates developed resistance to C/T; success may have been due to use of combination tobramycin.

^e^Patient experienced a recurrent infection after the first course of C/T which was cured by a second course of C/T.

^f^Following 6 weeks of treatment, cultures grew PsA resistant to C/T. Patient died 8 months after transition to palliative care.

BSI: Bloodstream infection; CI: Continuous infusion; CR: Carbapenem-resistant; cSSTI: Complicated skin and soft tissue infection; C/T: Ceftolozane/tazobactam; ESBL: Extended-spectrum β-lactamase; HCAP: Healthcare-associated pneumonia; ICU: Intensive care unit; IMC: Immunocompromised; LVAD: Left-ventricular assist device; MDR: Multidrug-resistant; NP: Nosocomial pneumonia; PDR: Pandrug-resistant; PsA: *Pseudomonas aeruginosa*; RTI: Respiratory tract infection; SSTI: Skin and soft tissue infection; UK: United Kingdom; US: United States; UTI: Urinary tract infection; VABP: Ventilator-associated bacterial pneumonia; XDR: Extensively-drug-resistant.
